# Sustainable Sensing with Paper Microfluidics: Applications in Health, Environment, and Food Safety

**DOI:** 10.3390/bios14060300

**Published:** 2024-06-07

**Authors:** Sanjay Kumar, Jyoti Bala Kaushal, Heow Pueh Lee

**Affiliations:** 1 Durham School of Architectural Engineering and Construction, University of Nebraska-Lincoln, Scott Campus, Omaha, NE 68182-0816, USA; 2 Department of Biochemistry and Molecular Biology, University of Nebraska Medical Center, Omaha, NE 68198, USA; 3 Department of Mechanical Engineering, National University of Singapore, 9 Engineering Drive 1, Singapore 117575, Singapore; mpeleehp@nus.edu.sg

**Keywords:** paper microfluidics, biosensors, healthcare, wearable sensors, environmental monitoring, food safety

## Abstract

This manuscript offers a concise overview of paper microfluidics, emphasizing its sustainable sensing applications in healthcare, environmental monitoring, and food safety. Researchers have developed innovative sensing platforms for detecting pathogens, pollutants, and contaminants by leveraging the paper’s unique properties, such as biodegradability and affordability. These portable, low-cost sensors facilitate rapid diagnostics and on-site analysis, making them invaluable tools for resource-limited settings. This review discusses the fabrication techniques, principles, and applications of paper microfluidics, showcasing its potential to address pressing challenges and enhance human health and environmental sustainability.

## 1. Introduction

In the past few decades, the increasing demand for portable, cost-effective, and environmentally friendly sensing technologies has driven the rapid advancement of paper-based microfluidics. Leveraging the inherent properties of paper, such as its low cost, biocompatibility, and ease of fabrication, researchers have developed innovative sensing platforms capable of performing complex analytical tasks with minimal resources.

One of the most compelling aspects of paper-based biosensors is their ability to meet the ASSURED criteria outlined by the World Health Organization (WHO) for point-of-care testing. These criteria emphasize the importance of tests being affordable, sensitive, specific, user-friendly, rapid and robust, equipment-free, and deliverable to those in need [1,2,3]. The paper microfluidics concepts are prominently utilized in healthcare, where it has revolutionized diagnostic testing, particularly in resource-limited settings. By integrating various biochemical assays and detection methods onto paper substrates, clinicians can now perform rapid and accurate diagnoses of various diseases, ranging from infectious diseases like malaria and HIV to chronic conditions such as diabetes and cancer. Moreover, the simplicity and portability of paper-based diagnostic devices make them well-suited for decentralized healthcare delivery, enabling point-of-care testing in remote or underserved communities.

Beyond healthcare, paper microfluidics plays a crucial role in environmental monitoring by providing cost-effective solutions for detecting pollutants and contaminants in air, water, and soil. By functionalizing paper with specific reagents or sensors, researchers can develop portable devices capable of detecting various environmental pollutants, including heavy metals, pesticides, and toxic gases. These paper-based sensors offer real-time monitoring capabilities and can be deployed in field settings to assess environmental quality and identify potential hazards.

Additionally, paper microfluidics contributes significantly to food safety and security by enabling the rapid and on-site detection of foodborne pathogens, adulterants, and contaminants. With the global food supply chain becoming increasingly complex and vulnerable to contamination, there is a growing need for robust and cost-effective methods to ensure the safety and integrity of food products. Paper-based sensors offer a promising solution by providing rapid, sensitive, and user-friendly tools for detecting harmful substances in food samples, helping prevent foodborne illnesses, and mitigating economic losses.

In summary, sustainable sensing with paper microfluidics holds immense promise for addressing critical challenges in healthcare, environmental monitoring, and food safety. Leveraging the unique attributes of paper, researchers are continuously innovating and introducing new sensing platforms that have wide-ranging implications for enhancing human well-being and preserving the environment. This manuscript provides a thorough overview of the current advancements in paper-based sensing technologies and their diverse health, environmental, and food-contaminant-detection applications. This comprehensive review is a valuable resource for researchers, practitioners, and enthusiasts in microfluidics, biotechnology, and environmental science, offering insights into the current state and future directions of biodegradable paper microsystems for health and environmental applications.

## 2. Fundamentals of Paper Microfluidics

### 2.1. Paper Types and Their Characteristics

Paper-based sensors leverage a wide variety of paper substrates, such as filter papers [4,5,6], nitrocellulose membranes [7,8,9,10], office papers (70–180 gsm) [11,12,13,14], tissue paper [15], photo (e.g., glossy) papers [16,17], waterproof papers [18], polyester papers [19], flexible polyethylene naphthalate sheets [20,21], and chromatography paper [22,23]. Notably, Whatman brand chromatography papers are among the most extensively utilized choices. This preference stems from the exceptional wicking ability that Whatman papers exhibit. Whatman offers a range of fibrous filter papers, such as Grade 1 to Grade 4, each characterized by distinct properties that find applications in various qualitative analytical techniques. These applications span general laboratory filtration, qualitative air pollution monitoring, soil analysis, food testing, and more. Whatman Filter Paper Grade 1, widely utilized in laboratory filtration, is renowned for its superior fine particle retention and rapid filtration capabilities. Composed of high-quality cellulose fibers, this filter paper basis weighs 88 g/m^2^, with a nominal particle retention rating of around 11 μm, making it highly effective in separating very fine particles. With a moderate thickness of 180 μm and a porosity of 10.5 s, it balances quick filtration and efficient particle retention. Whatman Filter Paper Grade 2, another well-established filtration medium, is recognized for its fine particle retention and moderate flow rate. It is manufactured from high-quality cellulose fibers weighing 103 g/m^2^ and offers a nominal particle retention of approximately 8 μm. Its balanced construction, with increased thickness (190 μm) compared to Grade 1, ensures efficient particle retention while allowing relatively faster filtration. Whatman Filter Paper Grade 3, made from high-quality alpha cotton cellulose (basis weight of 187 g/m^2^), provides a nominal particle retention of approximately 6 μm, with moderate thickness (309 μm) and a porosity of 26 s. Finally, Whatman Filter Paper Grade 4, designed for robust and versatile filtration, is crafted from high-quality alpha cotton cellulose, offering a nominal particle retention of approximately 20–25 μm, with moderate thickness (205 μm) and basis weight of 92 g/m^2^.

Nitrocellulose membrane papers, derived from the nitration of cellulose, are integral components in laboratory techniques such as Western blotting and immunoassays. Renowned for their highly porous structure of 0.45 μm and 12 μm pore sizes, these membranes provide efficient protein binding, ensuring a large surface area for immobilization. Their uniform pore size distribution guarantees consistent and reproducible outcomes, while their hydrophobic nature facilitates the transfer of hydrophobic proteins during blotting. With a high binding capacity, compatibility with various immunodetection methods, and versatility for protein and nucleic acid applications, nitrocellulose membrane papers play a crucial role in molecular biology and biochemistry, offering purity and reliability in experimental procedures.

Tissue paper is a versatile and widely used material characterized by its random packing of cellulose microfibers. These microfibers, ranging from several hundred micrometers in length to 50–100 μm in diameter, contribute to tissue paper’s softness, absorbency, and strength. Derived from wood pulp or plant-based sources, tissue paper is known for its comfort and durability, making it suitable for facial tissues, toilet paper, and napkins. However, it has been used in research applications such as oil/water separation, wearable sensors, etc. [24].

Glossy paper, traditionally associated with printing and photography, has emerged as a subject of study for paper-based sensors due to its unique composition. Comprising cellulose fibers and inorganic fillers intricately blended into the paper matrix, glossy paper offers distinct advantages in sensor development. For example, Arena et al. [25] explored the use of glossy paper to create a flexible paper-based sensing device specifically designed for detecting ethanol. Unlike traditional filter paper, glossy paper’s surface properties are more amenable to modification, providing greater sensor design and customization flexibility. This shift to glossy paper represents a novel approach, capitalizing on its composition to enhance the performance and adaptability of paper-based sensors, thereby expanding the scope of potential applications in analytical and diagnostic fields.

**Figure 1:**
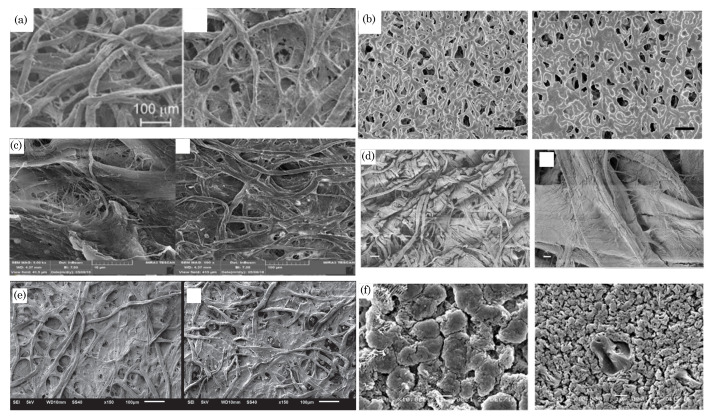
Scanning electron microscopy (SEM) images of different paper substrates. (**a**) Filter paper (Whatman Grade 1) (without treatment and with heat treatment at 600 °C). Reprinted with permission from Jiang et al. [26]. ©2015 The Authors, licensed under a Creative Commons Attribution 4.0 International License (CC BY-NC 4.0). (**b**) NC membrane (untreated and polyurethane acrylate-treated). Reprinted with permission from Lin et al. [27]. ©2022 The Authors, licensed under a Creative Commons Attribution 4.0 International License. Published by Springer Nature Limited. (**c**) Office paper at 10× and 100× magnification. Adapted from Jabar et al. [28] ©2019. Laser. (**d**) Native tissue paper (overview and close view). Reprinted with permission from Cao et al. [24]. ©2017 The Authors, licensed under a Creative Commons Attribution 4.0 International License. (**e**) Chromatographic paper at 150× (untreated and graphene oxide-modified), adapted from Fernandes et al. [29]. ©2019 The Authors, licensed under a Creative Commons Attribution License. Published by Sociedade Brasileira de Química. (**f**) Polyethylene terephthalate (PET) membrane. Adapted from Arahman et al. [30]. ©2017 The Authors, open access article distributed under the terms of the Creative Commons Attribution License.

Chromatography paper, composed primarily of high-quality cellulose fibers, is designed with specific technical specifications to facilitate the efficient separation and analysis of substance mixtures. Chromatography paper is available in different formats, such as sheets or rolls, tailored to specific chromatographic techniques, making it an essential tool for diverse analytical applications. Figure 1 shows the morphology of different paper substrates.

### 2.2. Paper Selection Factors

The choice of paper substrate in biosensing relies on several characteristics, including the capillary flow time (the time for the liquid sample to flow through lateral pores), paper thickness, pore size, porosity (percentage of air in the porous structure), and surface quality.

In paper-based microfluidic devices, capillary flow time refers to the duration it takes for a liquid sample to travel through the lateral pores of the paper substrate. The capillary flow time is inversely related to the capillary flow rate and expressed as s cm^−1^. It plays a vital role in defining the velocity and effectiveness of fluid movement within the microfluidic system. Specifically, the capillary flow time holds significant importance in creating paper-based diagnostic devices, especially in applications like lateral flow assays. Assessing the capillary flow time is instrumental in determining the optimal placement of the test and control lines on the nitrocellulose (NC) membrane.

The thickness of the paper substrate is an important parameter influencing the design and performance of paper-based microfluidic devices. It directly affects the capillary action, fluid flow dynamics, and device functionality. Thicker papers may impede the capillary flow, extending the path length for liquid absorption and affecting the speed of fluid traversal through paper channels. Moreover, paper thickness impacts sample absorption, with thinner papers potentially having a lower sample retention capacity, influencing device sensitivity and detection limits. The mechanical strength and integrity of the paper are also thickness-dependent, where thicker papers contribute to enhanced device durability. However, thicker papers may present challenges in fabrication processes such as cutting, printing, or folding, necessitating consideration of compatibility with chosen techniques. Consequently, optimizing paper thickness involves a delicate balance, requiring careful selection based on the specific needs of the intended microfluidic application.

Pore size is a pivotal parameter in paper microfluidics, exerting a profound impact on the performance and capabilities of microfluidic devices. Acting as conduits for capillary flow, the pores within the paper substrate guide fluid movement throughout the device. The capillary action hinges on pore size, with smaller pores facilitating slower yet controlled fluid flow, while larger pores allow for faster capillary flow. The speed and efficiency of fluid transport within the paper substrate are directly influenced by pore size. Fine-tuning this parameter is critical for optimizing fluid dynamics, ensuring precise sample movement to various device regions. Moreover, pore size governs the volume of sample absorption by the paper, providing customizable control over the sample absorption capacity to meet specific diagnostic or analytical needs. Pore size becomes pivotal in applications necessitating analyte separation, such as chromatographic assays. Varied analytes interact differently with the paper matrix, and adjusting the pore size enables selective separation. The resolution and sensitivity of paper-based assays are intricately tied to pore size, where smaller pores enhance resolution but may impede fluid transport speed. Striking a balance between these factors is essential for attaining paper microfluidic devices’ desired sensitivity and resolution.

Porosity refers to the percentage of air present in the porous structure of the paper substrate. It is a crucial parameter influencing the capillary action and fluid flow dynamics within microfluidic devices. The porosity of the paper directly impacts the movement of fluids through its pores. A higher porosity generally allows for better capillary flow, as there is more interconnected space for the fluid to travel. However, excessively high porosity may lead to rapid fluid flow and reduced control over the movement, potentially affecting the performance of the microfluidic device.

Permeability refers to the ability of the paper substrate to allow the flow of fluids through its structure. It is a crucial parameter influencing the capillary action and fluid transport dynamics within microfluidic devices. The permeability of the paper substrate determines how readily and efficiently liquids can traverse through its porous structure. A paper substrate with high permeability allows for rapid capillary flow, enabling the swift movement of fluids along the paper channels. On the other hand, lower permeability may result in slower capillary flow. The permeability of the paper is influenced by factors such as pore size, porosity, and the overall structure of the paper matrix. It is an important consideration in designing and optimizing paper-based microfluidic devices, particularly in applications where the precise control of fluid flow and transport dynamics is essential. For porous materials consisting of fibers with a circular cross-section of radius $r_{f}$, permeability can be approximated using the following empirical relation [31]:

(1) $$k=r_{f}^{2}\;\frac{\pi \varphi {\left(1-\sqrt{1-\varphi }\right)}^{2}}{24{\left(1-\varphi \right)}^{3/2}}$$

For random fibrous media, the permeability can be determined using the following correlation between permeability and porosity [32]:

(2) $$k=C_{1}r_{f}^{2}\;{\left(\sqrt{\frac{1-\varphi_{c}}{1-\varphi }-1}\right)}^{C_{2}}$$

Here, $\varphi_{c}$ represents the critical porosity value required for permeating flow, often referred to as the percolation threshold. The parameters *C*_1_ and *C*_2_ are associated with the network’s geometry.

Furthermore, the Kozeny–Carman equation can be employed to predict the permeability of granular isotropic porous materials, such as nitrocellulose membranes [33]:

(3) $$k=\frac{d^{2}\varphi^{3}}{180{\left(1-\varphi \right)}^{2}}$$

Here, d represents the average pore diameter, and porosity φ was determined empirically by measuring the volume of liquid absorbed by the materials [34].

### 2.3. Principles of Fluid Transport in Paper

Paper microfluidics operates on fluid flow without external forces, relying on capillary action to drive passive fluid movement through the paper substrate. The paper and the fluid’s contact surface interplay involves cohesive and adhesive forces. Interactions occur within the liquid at the liquid–air interface (cohesion) and between the solid–liquid interfaces (adhesion). The adhesive force facilitates the liquid’s spreading onto the porous substrate, while cohesive forces, such as surface tension, work to reduce the liquid–air interface’s area. Fluid flow occurs when the effect of adhesion surpasses that of cohesion. The wicking process is influenced by various physical and geometrical properties of porous media, including the paper material, paper structure, pore size, permeability, paper size and shape, and the physical properties of the liquid. Fluid transport can generally be classified into the wet-out process and fully wetted flow [35]. In the wet-out process, the fluid front wicks along the dry porous media and can be modeled using the classical Lucas–Washburn equation. Conversely, fluid transport occurs along the wetted porous media in fully wetted flow and is governed by Darcy’s law.

#### 2.3.1. Classical Lucas–Washburn Equation (Capillary Flow)

(4) $$l\left(t\right)=2\sqrt{\frac{k\gamma \cos\theta }{\Phi \mu r_{a}}}\sqrt{t}$$

where l(t) denotes the length of the wetted region of the paper at time *t*; *k* represents the permeability of the paper, reflecting how readily fluid can traverse a specific paper substrate and contingent on pore size and geometry; μ signifies fluid viscosity; γ represents the interfacial surface tension of the liquid; $r_{a}$ is the average pore radius; and *t* stands for the liquid penetration time. The Lucas–Washburn equation can be formulated in the following manner by incorporating the influence of tortuosity on capillary flow:

(5) $$l\left(t\right)=2\sqrt{\frac{r_{a}\gamma \cos\theta }{2\mu \tau^{2}}}\sqrt{t}$$

In this context, tortuosity is defined as $\tau ={\left(L_{e}/L\right)}^{2}$, where $L_{e}$ represents the effective path length between two intermittent points in the liquid and *L* is the straight path length. The parameter τ consistently holds a value greater than one.

#### 2.3.2. Darcy’s Law for Fluid Flow

Darcy’s Law is a fundamental equation describing fluid flow through porous media, and it applies to various contexts, including paper microfluidics. In this context, the imbibition rate of the fluid $\hat{u}$ through the paper substrate can be determined by Darcy’s law, as per the following equation:

(6) $$\hat{u}=\frac{k_{i}\Delta P}{\mu l(t)}$$

In the given expression, $k_{i}=k/\varphi$ represents the interstitial permeability of the paper strip and $\varphi =1-\frac{m}{\rho_{c}h}$ is the porosity of the medium [36]. Other variables include m, which denotes the basis weight; $\rho_{c}$ and h representing the density and thickness of the porous substrate, respectively; and ΔP, which stands for the pressure difference over the wetted region, often referred to as Laplace pressure.

For a straight paper strip device, Darcy’s law for fluid flow can be modified as follows:

(7) $$Q=-A\cdot k\cdot \frac{\Delta P}{L}$$

*Q* represents the volumetric flow rate of the liquid; A (equal to w×h) signifies the cross-sectional area of the paper; k stands for the permeability of the paper, which measures the ease of fluid flow through the paper substrate; and ΔP=P(0)−P(L) indicates the pressure drop across the paper, with *P*(0) denoting the pressure at *x* = 0 and *P*(*L*) representing the average capillary pressure at the fluid front. *L* is the length of the paper. This equation introduces a negative sign to consider the flow occurring in the direction of decreasing pressure. Additionally, the term $\frac{\mu L}{kwh}$ is defined as the flow resistance ($R_{hyd}$):

(8) $$Q=-\frac{\Delta P}{R_{hyd}}$$

Equation (8) bears similarity to Ohm’s law in an electric circuit, expressed as ΔV=RI, where *I* denotes the electric current, *R* is the electrical resistance, and ΔV represents the potential drop. In hydrodynamic systems, the volumetric flow rate *Q* signifies the volume per unit time, while in an electric system, the current represents the charge per unit time. Moreover, ΔP (energy per volume) draws an analogy to potential drop (energy per charge).

Moreover, beyond the conventional linear channels in paper strips, researchers have introduced a variety of configurations to control fluid transport, each characterized by distinct dynamics attributed to shapes like circular, trapezoidal, sector-shaped, multisection medium, and other arbitrary geometries [37,38,39,40,41]. For an in-depth exploration of these geometries, readers can refer to the comprehensive summary provided by Kumar et al. [42].

### 2.4. Dimensionless Numbers for Fluid Transport

In paper microfluidics, the intricate phenomena of fluid flow can be effectively characterized and understood by employing a series of dimensionless numbers [43]. These dimensionless numbers play a crucial role in delineating the relative significance of different physical factors governing the behavior of fluids within the porous paper substrate. Dimensionless numbers are fundamental in scaling and normalizing various parameters, enabling researchers and engineers to draw meaningful comparisons and insights across different systems and scales.

#### 2.4.1. Capillary Number (*Ca*)

It is defined as the ratio of viscous forces to capillary forces and is expressed as

(9) $$Ca=\frac{U\mu }{\gamma }$$

Here, *U* (m/s) represents the velocity of the flow, γ (N/m) denotes the surface tension at the water/paper interface, and μ (kg/(ms)) stands for the viscosity of the fluid.

The capillary number helps to characterize the dominance of capillary forces over viscous forces in a given system. In the context of paper microfluidics, it provides insights into the ability of capillary action to drive fluid flow through the paper substrate. When Ca is small, viscous forces dominate, and the flow is slow and controlled by viscosity. On the other hand, when Ca is large, capillary forces take precedence, resulting in faster, capillary-driven flow. A low capillary number is often desirable for paper-based devices, ensuring controlled and predictable fluid flow. Understanding and manipulating Ca is essential for designing effective paper microfluidic devices, especially in point-of-care diagnostics and environmental monitoring applications.

#### 2.4.2. Reynolds Number (*Re*)

A dimensionless quantity characterizes the relative importance of inertial forces to viscous forces in fluid flow. In paper microfluidics, the Reynolds number helps assess the nature of fluid flow through the porous substrate. It is defined as

(10) $$Re=\frac{\rho UL}{\mu }$$

where ρ is the fluid density, *U* is the characteristic velocity of the flow, *L* is a characteristic length (e.g., pore size, channel width), and μ is the dynamic viscosity of the fluid.

The Reynolds number classifies flow regimes into laminar and turbulent. In paper microfluidics, where flow is typically slow and capillary-driven, the flows are often in the laminar regime (low Re). Laminar flow is characterized by smooth and predictable streamlines, making it suitable for the controlled transport of fluids within microchannels or porous media. In a porous medium, if the Re is less than one, the flow is characterized as laminar and linear, and Darcy’s Law is applicable. However, when Re exceeds 10, the flow remains laminar but is no longer linear. In this regime, inertial forces become significant, causing a departure from the linear behavior, and consequently, Darcy’s Law is no longer valid [43,44].

#### 2.4.3. Weber Number (*We*)

The Weber number (*We*) is a dimensionless parameter that characterizes the ratio of inertial forces to surface tension forces in a fluid flow. It is particularly relevant in understanding the deformation and breakup of liquid droplets. The Weber number is defined as

(11) $$We=\frac{\rho U^{2}L}{\sigma }$$

where ρ is the fluid density, *U* is the characteristic velocity of the flow, *L* is a characteristic length (e.g., pore size and channel width), and σ is the surface tension at the liquid–air interface.

The Weber number indicates the dominance of inertial forces over surface tension forces. A low Weber number is often desirable in paper microfluidics, where capillary action and surface tension are crucial in fluid transport through porous substrates. A low We signifies that capillary forces and surface tension are sufficient to overcome inertial forces, allowing for stable and controlled fluid flow.

#### 2.4.4. Schmidt Number (*Sc*)

The Schmidt number (*Sc*) is a dimensionless quantity that characterizes the relative importance of momentum and mass transport in the fluid flow. It is defined as the ratio of the kinematic viscosity of the fluid to its mass diffusivity:

(12) $$Sc=\frac{\mu }{\rho \;D}$$

Here, ν=μ/ρ is the kinematic viscosity (m^2^ s^−1^) and *D* is the is the mass diffusivity (m^2^ s^−1^).

Sc plays a crucial role in determining the effectiveness of mass transport processes, especially in cases involving simultaneous flow and diffusion. A high Schmidt number suggests that the diffusional transport of mass is dominant compared to the convective transport by fluid flow. Conversely, a low Schmidt number indicates that convective transport prevails over diffusional processes. Controlling mass transport is essential for applications such as chemical reactions, analyte detection, and other biological or chemical processes in paper microfluidic devices.

#### 2.4.5. *Péclet* Number ($Pe_{L}$)

The *Péclet* number ($Pe_{L}$) is a dimensionless quantity that characterizes the relative importance of convective transport to diffusive transport in a fluid flow system. It is defined as the ratio of the characteristic time of convective transport to the characteristic time of diffusive transport:

(13) $$Pe_{L}=Re\times Sc=\frac{U}{D/L}$$

The significance of the *Péclet* number lies in its ability to provide insights into the dominance of convective or diffusive transport mechanisms. A high $Pe_{L}$ indicates that convective transport is dominant, suggesting that fluid flow is crucial in transporting species within the porous medium. On the other hand, a low $Pe_{L}$ means that diffusive transport is more significant, indicating that the concentration gradient is the primary driving force for mass transport.

For applications in paper microfluidics, such as chemical reactions, analyte detection, or biological assays, understanding the $Pe_{L}$ number is crucial for optimizing the design and performance of the devices. Balancing convective and diffusive transport is essential to ensure efficient and controlled mass transport, ultimately influencing the accuracy and reliability of the processes carried out in paper microfluidic systems.

## 3. Classifications of Paper-Based Assays

Paper-based sensors are versatile diagnostic tools that utilize the properties of paper to detect various analytes. These sensors fall into three main classifications: dipstick tests, lateral flow assays (LFAs), and microfluidic paper-based analytical devices (μPADs).

### 3.1. Dipstick Assays

**Figure 2:**
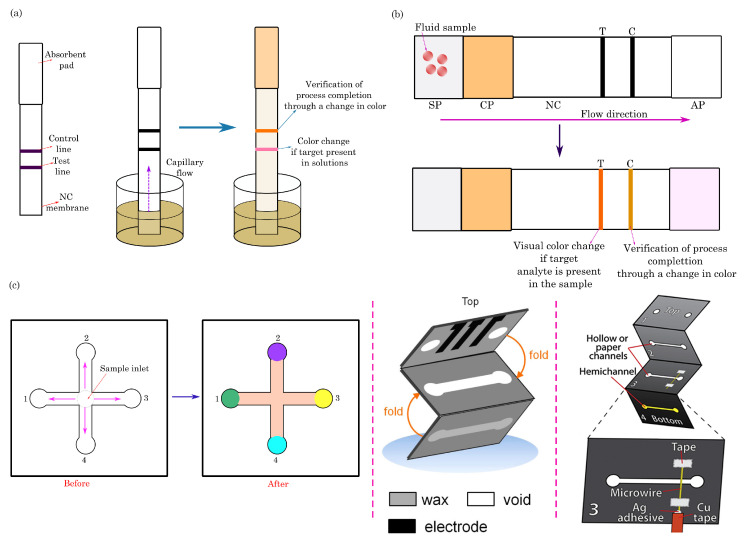
Schematics illustrating various paper-based assays: (**a**) Dipsticks typically comprise the test line and control line printed on the NC membrane, with an absorbent pad (crafted from filter paper) to soak up excess sample fluid. (**b**) Lateral flow assays (LFAs) encompass key components such as SP: sample pad, CP: conjugate pad, NC: nitrocellulose membrane, T: test line, C: control line, and AP: absorbent pad. (**c**) Microfluidic paper-based analytical devices (μPADs): a simple patterned paper device designed for multianalyte detection (left), origami-based 3D μPADs with hollow channels (center and right). Reprinted with permission from Renault et al. [47]. ©2014 American Chemical Society and from Carrell et al. [48]. ©2019 The Authors, under a Creative Commons license CC BY-NC-ND-4.0, Elsevier B.V.

Dipstick test strips consist of paper pads with dried capture reagents affixed to a supporting plastic strip. A paper strip is immersed into a liquid sample in the dipstick assay process. The sample traverses the strip, interacting with specific reagents immobilized, resulting in a discernible signal at the test line (Figure 2a). The intensity or shade of the produced color in dipstick assays sometimes enables users to estimate the approximate or semi-quantitative concentration of the analyte. Key advantages of dipstick assays include their simplicity, cost-effectiveness, and the capability to test for multiple analytes simultaneously. However, dipstick assays often exhibit drawbacks such as poor detection limits and limited specificity [45,46].

### 3.2. Lateral-Flow Assays

Paper-based lateral flow assays (LFAs) consist of overlapping paper substrates, including a sample pad (SP) for receiving the liquid sample, a conjugate pad (CP) with labeled reagents (e.g., colloidal gold particles), a nitrocellulose (NC) membrane containing test and control lines, and an absorbent pad (AP) to soak up excess sample fluid (Figure 2b). The working principle involves applying the sample to the pad, initiating capillary flow that guides the sample through the conjugate pad, which interacts with the labeled reagents. The sample then traverses the nitrocellulose membrane, binding to immobilized capture agents at the test line if the target analyte is present, forming a visible line. The control line, containing immobilized capture agents for validation, always produces a line. The absorbent pad at the end facilitates liquid flow. This assay allows for rapid on-site detection, with the presence or absence of lines providing a visual interpretation of results, and its versatility makes it valuable for diagnostics and point-of-care testing.

### 3.3. Microfluidic Paper-Based Analytical Devices (μPADs)

Paper-based microfluidic biosensors (μPADs) represent an innovative class of diagnostic tools that integrate microfluidic channels on paper [49]. These biosensors leverage the capillary action of paper to control the flow of liquids through predefined channels. Microfluidic components enable the precise manipulation of samples and reagents, enhancing the sensitivity and specificity of assays. By incorporating various detection zones on the paper, μPADs can be customized to detect multiple analytes simultaneously (Figure 2c).

These paper-based assays are presently employed across diverse applications for detecting diseases, pathogens, toxins, pollutants, food safety, and, most notably, in the recent context, for detecting COVID-19 [50,51,52]. A detailed discussion on applications of these assays is discussed in Section 7 and Section 8.

## 4. Fabrication Techniques for Paper-Based Devices

Paper-based microfluidic devices have gained popularity due to their simplicity, cost-effectiveness, and ease of fabrication. Several techniques are employed to fabricate these devices, each offering unique advantages. The manufacturing processes for paper microfluidics involve making specific sections of the paper hydrophilic to enable smooth sample flow, while other sections are made hydrophobic to form the channel walls. Broadly, fabrication methods can be categorized into two approaches: the first involves treating hydrophilic paper with hydrophobic materials to shape the desired channels, while the second approach entails cutting the paper using various tools such as knives or lasers to generate the channel pattern. Here are some commonly used fabrication techniques for paper-based microfluidic devices:

### 4.1. Blade Cutting/Plotting

Blade cutting or plotting is a versatile method providing a straightforward and precise means of creating desired patterns and structures. This technique involves using a cutting or plotting machine equipped with a sharp blade to cut through paper substrates precisely, shaping them according to a predefined digital design.

The process begins with creating a digital design or blueprint of the intended paper-based device using design software. This digital file guides the cutting or plotting machine, detailing the specific features, dimensions, and patterns. The paper substrate, typically selected for its compatibility with blade cutting, is then securely fixed onto the machine’s work surface. The machine is calibrated to accommodate the specific properties of the paper and the design requirements. Adjustments to parameters such as blade depth, speed, and cutting force are made to ensure accurate and clean cuts. The digital design file is loaded into the cutting or plotting machine, specifying the desired cutting settings. The cutting or plotting machine, guided by the digital design, moves the sharp blade across the paper substrate, accurately cutting or scoring along the defined lines. The process is precise and repeatable, allowing for the creation of intricate patterns, microfluidic channels, or other features.

Blade cutting/plottings offer advantages such as simplicity, cost-effectiveness, and quick turnaround times. However, it may have limitations in achieving excellent features or complex geometries compared to more advanced fabrication techniques. Nonetheless, it remains a popular choice for rapid prototyping and producing paper-based devices for various applications.

### 4.2. Laser Cutting

**Figure 3:**
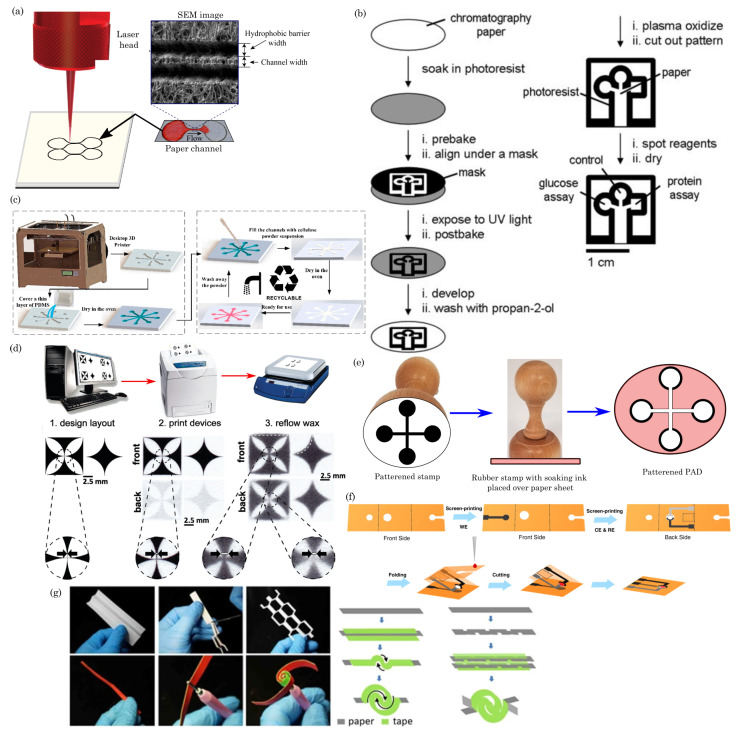
Schematics of various fabrication techniques for paper-based sensors. (**a**) Laser cutting, reproduced with permission from Mahmud et al. [53]. ©2018 The Authors, MDPI (Basel, Switzerland). (**b**) Photolithography, reprinted with permission from Martinez et al. [54]. ©2007 John Wiley & Sons, Inc. (**c**) 3D printing, reprinted with permission from He et al. [55]. ©2016 The Authors, published by MDPI, 2016. (**d**) Wax printing, reprinted with permission from Carrilho et al. [56]. ©2009 American Chemical Society. (**e**) Patterning paper using the stamping method. (**f**) Design and fabrication process of the origami-paper-based device. Reprinted with permission from Wang et al. [57]. Licensed under a Creative Commons Attribution 4.0 International License, ©2024 Springer Nature Limited. (**g**) Images and schematic representation depicting the process of crafting 2D and 3D vertical paper analytical devices (vPADs) through the utilization of quilling and kirigami principles. Reprinted with permission from Gao et al. [58]. ©The Author(s), licensed under a Creative Commons Attribution 4.0 International License.

Laser cutting, a meticulous and versatile method for fabricating paper-based devices, utilizes a laser beam to intricately cut or engrave patterns, channels, and features into paper substrates, resulting in well-defined structures. The process involves several key steps, including creating a digital design or blueprint using design software. This digital file serves as a guide for the laser-cutting process, detailing specific features and dimensions. Material selection is crucial, with the chosen paper substrate needing the right thickness and properties to achieve precise cuts without excessive burning. Calibration of the laser cutter ensures alignment with paper and design specifications, adjusting settings such as the laser power, speed, and focus. Once the digital file is loaded into the laser-cutting machine, the process is initiated, and the laser cutter faithfully follows the programmed path to cut through the paper substrate. The high-energy laser beam vaporizes or burns away the material along the designated cutting lines. Figure 3a depicts the conventional laser-cutting method for fabricating paper devices. Laser cutting offers remarkable precision, minimal material wastage, and the capability to craft intricate and personalized designs, making it ideal for applications like paper-based microfluidic devices and sensors requiring precise and detailed structural features.

### 4.3. Photolithography

Photolithography is a sophisticated technique employed in fabricating paper-based devices, enabling the creation of intricate patterns and microscale features. The process involves several sequential steps, beginning with preparing a photoresist-coated substrate. The chosen paper substrate is coated with a light-sensitive photoresist material, forming a uniform layer. A photomask containing the desired pattern is then placed near the coated substrate. Exposure to ultraviolet (UV) light passes through the transparent regions of the photomask, initiating a chemical reaction in the photoresist. The exposed areas become either more or less soluble, depending on the type of photoresist used. After exposure, the substrate undergoes a development process, where a solvent is applied to remove the soluble portions of the photoresist. This reveals the pattern on the substrate corresponding to the photomask. The developed substrate is subjected to additional treatments, such as baking, to enhance the pattern’s stability. The exposed and developed paper substrate can now act as a template for creating hydrophobic barriers, fluidic channels, or other functional elements. Various methods, such as wax printing or plasma treatment, can selectively modify the paper’s properties. Figure 3b illustrates the step-by-step photolithography methods for patterning paper devices.

Photolithography offers high precision and resolution, making it suitable for applications that require intricate designs and well-defined microstructures. However, it may involve using specialized equipment and chemicals, adding complexity to the fabrication process.

### 4.4. 3D Printing

3D printing, or additive manufacturing, is a cutting-edge technique in fabricating paper-based devices. This method enables the creation of three-dimensional structures layer by layer, providing precise control over design and geometry. The 3D printing process involves utilizing digital design software to create a three-dimensional model of the intended paper-based device, serving as a blueprint for the 3D printer. Material selection is crucial, with biodegradable and eco-friendly materials, such as specific polymers, commonly used for paper-based devices compatible with 3D printing.

To ensure precise layer deposition, the 3D printer is calibrated, adjusting parameters like layer thickness, print speed, and temperature based on the chosen material and design specifications. The digital file is loaded into the 3D printer, specifying the desired settings. The printer deposits layers of the selected material, building up the three-dimensional structure according to the digital model. This layer-by-layer approach allows for sophisticated designs and complex geometries, as illustrated in Figure 3c.

After the printing process is complete, any support structures used during printing are removed, and additional post-processing steps, such as sanding or coating, may be performed to refine the surface and enhance specific properties of the 3D-printed paper device. The overall 3D printing method offers advantages such as rapid prototyping, customization, and the ability to produce complex structures that may be challenging with traditional fabrication methods. However, careful consideration of the material selection, printer calibration, and post-processing steps is essential to optimize the performance and quality of 3D-printed paper-based devices.

### 4.5. Screen Printing

Screen printing is a versatile and economical technique for producing paper-based microfluidic devices. This method facilitates the application of hydrophobic barriers and functional inks onto porous paper substrates, thereby establishing fluidic channels with diverse applications, including diagnostics and chemical analysis. The fabrication begins with developing a digital design or stencil outlining the microfluidic channels, test zones, and additional features. Subsequently, the stencil or design is secured onto the mesh screen, aligning it with the intended microfluidic layout. The screen is then coated with a layer of hydrophobic or wax-based ink. Execution of the printing process involves placing the inked screen onto the paper substrate utilizing a squeegee to distribute the ink evenly across the screen. This action propels the ink through the mesh, deposits it onto the paper, and defines the desired pattern. The screen is lifted to unveil the printed design, and this procedure is iterated for each layer or color in the overall design. Thorough drying of the printed paper is imperative to ensure proper ink adherence and prevent smudging.

Depending on the ink formulation, specific devices may need post-printing treatments. One standard post-printing treatment involves subjecting the printed paper to heat, enhancing the hydrophobic properties of the ink, and ensuring the formation of effective barriers. This step optimizes the paper-based microfluidic device, aligning its performance with specific requirements.

### 4.6. Wax Printing

Wax printing relies on the hydrophobic properties of wax to create fluidic channels on paper. The process involves selectively depositing wax onto the paper substrate to define the boundaries of channels and hydrophilic zones. The hydrophobic wax barriers prevent liquid flow in certain areas, while the untreated paper remains hydrophilic, facilitating capillary-driven fluid transport.

The printing involves a multistage process. The desired fluidic channel pattern is designed using graphic design software. This layout defines the paper’s test zones, channels, and other features. The designed pattern is then printed onto the paper using a wax printer. The wax is typically melted and deposited onto the paper, creating hydrophobic barriers. Commonly used waxes include paraffin or a mixture of paraffin and other additives. After printing, the paper is heated to allow the wax to penetrate the paper fibers, enhancing its hydrophobic properties. This step ensures better control over fluid flow and prevents lateral spreading. The paper may be layered or folded to create three-dimensional structures, and additional materials, such as membranes or reagents, can be integrated at specific locations. Figure 3d describes the step-by-step wax printing methods for patterning paper devices.

### 4.7. Inkjet Printing

Inkjet printing emerges as a precise and versatile approach for crafting paper-based microfluidic devices, relying on the controlled deposition of liquid inks onto paper substrates. This method facilitates the generation of intricate patterns, microfluidic channels, and functional elements. The process entails several key steps: First, design the desired microfluidic layout, test zones, and other features using digital design software such as AutoCAD and CorelDRAW. The resulting digital file guides the inkjet printer in creating the specified patterns. Next, choose appropriate inks based on application requirements, which may include hydrophobic barriers, conductive materials, or biofunctional agents, depending on the intended purpose of the paper-based device. Calibrate the inkjet printer to ensure accurate and consistent droplet deposition, with crucial parameters such as droplet size, spacing, and positioning. Load the designed digital file into the inkjet printer, specifying the desired settings. The printer then dispenses tiny droplets of ink onto the paper surface based on the digital design, bringing the defined microfluidic features and patterns to life. The inkjet printing process may be repeated for complex designs with multiple layers or colors for each layer, necessitating precise alignment to achieve the intended device structure. Thorough drying of the printed paper is crucial to prevent ink smudging and ensure steadfast adherence to the printed features.

### 4.8. Embossing

Embossing is a technique that fabricates paper-based devices to create raised patterns or structures on a paper substrate. The process involves designing the desired pattern using digital design software such as AutoCAD and CorelDRAW. This pattern dictates the expanded features of the paper device. A heated metal die is chosen as the embossing material for the embossing process. The die, designed to match the intended pattern, transfers the pattern onto the paper. The paper substrate is prepared on a clean, flat surface. The metal die is heated to the required temperature. Heat is crucial in softening the paper fibers, enabling them to conform to the raised pattern on the die. The embossing process begins by positioning the heated die over the designated area on the paper. Pressure is applied to the die, pressing it onto the paper substrate. The combination of heat and pressure causes the paper fibers to deform, adopting the die pattern. The die is held for a specific duration to ensure proper embossing. After embossing, the paper is allowed to cool and set. This step is essential for the paper to retain the raised pattern effectively. A quality check inspects the embossed paper, ensuring the increased features are well-defined and consistent. Figure 3e shows the step-by-step process for the stamping method.

Depending on the application, additional post-processing steps may be undertaken. For instance, the embossed paper may be coated with hydrophobic substances to modify its fluid-handling properties. Embossing is particularly useful for creating three-dimensional structures on paper, such as microfluidic channels or detection zones. It is a relatively simple and cost-effective method, making it suitable for various applications in paper-based microfluidics and analytical devices.

### 4.9. Origami, Quilling, and Kirigami

Origami, quilling, and kirigami are innovative methods for fabricating paper-based devices, leveraging folding, quilling, and cutting principles to create intricate structures with diverse functionalities.

Origami, an ancient Japanese art form, involves precisely folding paper to create three-dimensional structures without cutting or adhesive. In paper device fabrication, origami provides an elegant means of constructing complex and functional designs. Researchers and engineers use origami techniques to fold paper into specific shapes, forming containers, channels, or dynamic components. The process typically begins with the design of a flat pattern that, when folded along predetermined lines, transforms into the desired 3D structure. The patterns are often created using computer-aided design (CAD) software. Origami-based paper devices have been developed for μPADS applications, such as creating fluidic channels and reservoirs through folding.

Quilling-based paper device fabrication involves creatively adapting quilling, a paper art form, to construct functional microfluidic devices [59]. This innovative approach utilizes the rolling, shaping, and arranging of paper strips to create intricate structures, including microfluidic channels, reservoirs, and other components essential for analytical or diagnostic purposes. The process includes designing and planning the device layout, selecting suitable paper types, preparing quilling strips, employing quilling techniques to form desired shapes, assembling the components, and integrating functional elements. This method provides a cost-effective and customizable way to prototype simple microfluidic devices, offering accessibility and creativity in fabricating paper-based analytical tools for educational, research, or point-of-care applications.

Kirigami, an extension of origami, introduces the element of cutting into the folding process. This method allows for more intricate and flexible designs by strategically incorporating cuts and folds. In paper device fabrication, kirigami enables the creation of structures that can expand, contract, or exhibit specific movements. Designers use kirigami to craft patterns that, when folded and cut, result in functional and dynamic paper-based devices. This technique is particularly advantageous for applications requiring mechanical actuation or shape-changing capabilities. Kirigami-based devices have been found to be useful in flexible electronics and biomedical devices.

Figure 3f,g illustrates schematic representations of origami, quilling, and kirigami techniques employed in fabricating paper devices. These methods offer simplicity, low cost, and the ability to create complex structures without advanced equipment. However, precision in folding and cutting is crucial to achieving the intended functionalities. These methods have garnered attention for their potential to develop innovative and accessible solutions for various technological applications.

Table 1 provides a comprehensive summary of various fabrication techniques, detailing their specific characteristics and applications for paper-based sensors. The table encompasses a range of methods, highlighting each technique’s unique features, advantages, and potential limitations. It serves as a valuable reference for understanding how different fabrication processes can be tailored to enhance the performance and functionality of paper-based sensors in diverse applications.

## 5. Detection Techniques

### 5.1. Colorimetric Sensing

**Figure 4:**
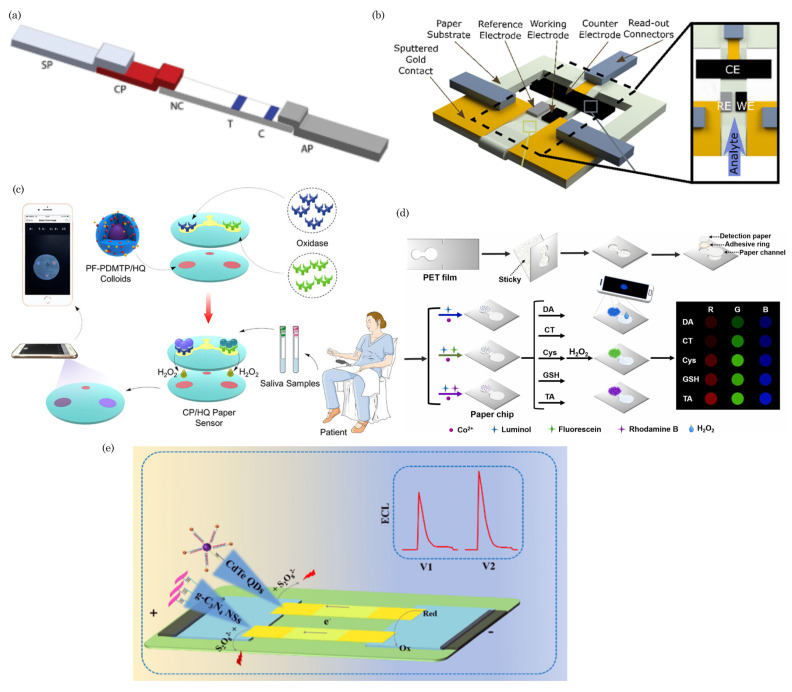
(**a**) Paper-based lateral flow assay for colorimetric sensing of dengue NS1. Reprinted with permission from Kumar et al. [109]. ©2018 AIP Publishing LLC. (**b**) Paper-based electrochemical sensors for glucose sensing, reprinted with permission from Valentine et al. [120]. ©2020 American Chemical Society. (**c**) Schematic diagram of the paper-based fluorescent sensor for rapid early screening of oral squamous cell carcinoma. Reprinted with permission from He et al. [121]. ©2023 American Chemical Society. (**d**) Paper-based chemiluminescence sensing of antioxidants (dopamine, CT, Cys, GSH, and TA): CL spectra (top) and CL images (bottom). Reprinted with permission from Li et al. [122]. ©2023 Elsevier B.V. (**e**) Illustration of a conceptual paper-based bipolar electrode electrochemiluminescence platform for detecting multiple miRNAs. Reprinted with permission from Wang et al. [123]. ©2020 American Chemical Society.

Colorimetric detection is widely used in paper-based microfluidic devices to analyze visual and quantitative data. Colorimetric sensing on paper-based devices operates on the principle of visual color change as an indicator of the presence and concentration of a specific analyte. Immobilized reagents on the paper matrix selectively react with the target substance, leading to a detectable color change upon interaction. The sample application allows the analyte to flow through the paper via capillary action, initiating various biochemical reactions, such as enzymatic reactions, antigen–antibody binding, or pH changes. The resulting color change is proportional to the analyte concentration, providing a simple and cost-effective means of on-site detection without the need for complex instruments. This approach is widely applied in medical diagnostics, environmental monitoring, and food safety, offering a user-friendly solution for rapid analyte quantification. Figure 4a shows schematics of the colorimetric sensing of dengue NS1 using a paper-based lateral flow assay [109]. In this assay, the sample is loaded onto the SP and migrates across the strip. At the CP, the NS1 antigen (Ag) interacts with immobilized Au-rGO-Ab conjugates, forming Au-rGO-Ab-Ag complexes. These complexes travel through the membrane via capillary action. At the test line, they bind to capture antibodies, creating a sandwich (Au-rGO-Ab-Ag-Ab) and producing a colored band. The absence of Ag results in no band. Excess-labeled antibody conjugates bind to secondary antibodies at the control line, creating another colored band and confirming assay completion. The absorbent pad absorbs the excess buffer and unbound nanoparticles.

### 5.2. Electrochemical Sensing

Electrochemical detection is a widely utilized method in paper-microfluidics-based sensors. It enhances their versatility and efficacy by leveraging electrochemical reactions at the sensor’s surface for target analyte detection and quantification. The integration of paper microfluidics, driven by capillary action facilitating fluid flow, seamlessly combines with electrochemical detection, resulting in efficient and portable sensing platforms. Key components of electrochemical sensors, including the working electrode (WE), reference electrode (RE), and counter electrode (CE), play crucial roles. The WE, typically made of conductive materials like carbon or metal, is the primary site for analyte electrochemical reactions, often modified for enhanced selectivity and sensitivity. The RE maintains a stable reference potential, accurately determining the electrochemical reaction at the working electrode. CE completes the electrical circuit by providing a pathway for the flow of electrons during the electrochemical reaction. It is often made of conductive materials such as platinum or graphite and is not directly involved in the analyte reaction.

In paper-based devices, these electrodes are embedded into the paper matrix. These electrodes facilitate electrochemical reactions during the sensing process. Reagents, such as enzymes or antibodies, immobilized on the electrodes selectively interact with the target analyte, initiating an electrochemical response. Upon sample introduction, the immobilized reagents induce electrochemical reactions, with changes in redox states, conductivity, or pH depending on the sensing mechanism. The resulting electrochemical changes are detected using instrumentation like a potentiostat, and the recorded signals indicate the presence and concentration of the target analyte. The quantified electrochemical signals offer a quantitative assessment through digital displays or data analysis software.

In Figure 4b, schematic diagrams of a paper-based electrochemical sensor are depicted. These sensors utilize electrochemical sensing electrodes created by drop-casting a carbon nanotube (CNT) suspension onto paper substrates with varying porosities. The fabrication process involves a combination of laser cutting, CNT solution drop-casting, and origami techniques to produce arrays of diagnostic devices. Laser cutting is employed to delineate the electrode sensing area, facilitating the straightforward drop-casting of the CNT suspension without needing a separate patterning process. Origami techniques are then utilized to establish connections between the working, reference, and counter electrodes with the electrolyte, enhancing the functionality and manufacturability of the device.

### 5.3. Fluorescence

Fluorescence detection is a robust and widely utilized method in paper-microfluidics-based sensors, offering high sensitivity and specificity for detecting various analytes [110,111,112,113,114]. This approach capitalizes on the innate fluorescence properties of specific molecules known as fluorophores, facilitating target substances’ precise identification and quantification.

In the typical configuration of μPADs, specialized reagents or probes contain fluorophores that exhibit selective interactions with the target analyte. When exposed to UV light, these probes undergo distinct fluorescence changes upon introducing a sample containing the target analyte. These changes may manifest as emission intensity or wavelength alterations, which can be detected and measured using a fluorescence imaging system. This imaging system allows for real-time monitoring and quantitative analysis, making it particularly valuable in medical diagnostics, environmental monitoring, and food safety applications. Integrating fluorescence detection into microfluidic paper-based sensors enhances their capabilities, providing a powerful tool for the rapid, on-site, and multiplexed detection of various analytes.

In Figure 4c, a schematic diagram illustrates the fabrication process and detection mechanism of a double-layered, paper-based fluorescent sensor. This sensor comprises an upper reaction layer containing two oxidases (lactate oxidase and choline oxidase) and a bottom fluorescent layer loaded with composite porphine-grafted fluorescent polymer colloids (PF-PDMTP/HQ). The sensor operates by detecting the significant and rapid decrease in fluorescence of porphine resulting from the oxidation reaction between saliva and the oxidases. This reaction is followed by fluorescence resonance energy transfer from oxidized hydroquinone. As a result, the developed fluorescent paper sensor enables the visual detection of oral squamous cell carcinoma (OSCC), which can be further confirmed through grayscale variation analysis using smartphone scanning.

### 5.4. Chemiluminescence

Chemiluminescence is a detection method commonly employed in paper-microfluidics-based sensors, offering a sensitive and versatile means of analyzing target analytes [115,116,117]. The fundamental principles of chemiluminescent sensing on paper-based devices involve immobilizing specific chemiluminescent reagents, such as enzymes or light-emitting molecules, onto the paper matrix. These reagents are selected for their ability to produce light upon interacting with the target analyte. Subsequently, the sample containing the analyte is applied to the paper surface, initiating a chemical reaction with the immobilized reagents and resulting in light emission. The chemiluminescent response releases energy through light, which is then detected and quantified using a photodetector or imaging system. The light emission’s intensity correlates with the analyte’s concentration, providing a quantitative readout that can be visualized through an imaging system or measured using specialized instrumentation like a photodetector.

Figure 4d illustrates a microfluidic paper chip-based multicolor chemiluminescence sensor designed to detect five antioxidants. This paper chip comprises four layers: a polyethylene terephthalate (PET) film, a paper channel, a double-sided adhesive ring, and a round-shaped detection paper. The paper channel includes a large sampling zone connected to a small sampling zone. These components are mass produced using a home craft cutter printer. The detection paper undergoes modification through the sequential addition of 5 μL of 1 mM Co^2+^ solution followed by 5 μL of 5 mM chemiluminescent (CL) reagent, which could be luminol, a mixture of luminol and fluorescein, or a mixture of luminol and rhodamine B. Subsequently, the PET substrate, paper channel, double-sided adhesive ring, and modified detection paper are assembled to create multilayer paper chips suitable for further experimentation.

### 5.5. Electrochemiluminescence

Electrochemiluminescence (ECL) represents a cutting-edge detection method seamlessly integrated into paper-microfluidics-based sensors, providing a robust and precise analytical tool for detecting target analytes [118,119]. This innovative approach synergistically combines electrochemical and luminescent principles to achieve heightened sensitivity and selectivity.

In an electrochemiluminescence-based paper microfluidic sensor, the device incorporates essential components such as electrodes and chemiluminescent reagents. The electrodes are pivotal in facilitating electrochemical reactions that generate species in excited states. These excited states subsequently release photons during relaxation, resulting in luminescence. The beauty of this method lies in its ability to leverage the controlled electrochemical reactions to induce luminescence, offering a precise and sensitive means of detecting analytes.

The detection mechanism within ECL-based paper microfluidic sensors revolves around measuring the emitted light. The intensity of the emitted light is directly correlated with the concentration of the target analyte present in the sample. This quantitative correlation enables the precise analysis and quantification of analytes, making ECL-based sensors invaluable in various applications, including medical diagnostics, environmental monitoring, and bioanalytical research. Also, integrating electrochemiluminescence into paper microfluidics enhances the analytical capabilities of these sensors and contributes to the development of portable, cost-effective, and efficient platforms for on-site detection. The sensitivity and selectivity achieved through ECL make it a promising technology for advancing point-of-care diagnostics and real-time monitoring in diverse fields.

Figure 4e depicts the schematic of a paper-based bipolar electrode electrochemiluminescence platform designed to detect multiple targets, specifically miRNA-155 and miRNA-126. In this setup, the electron transfer process in each bipolar electrode is electrically coupled with the electrochemiluminescence (ECL) reaction of each light-emitting probe due to the connection between the cathode and the anode. The DC power supply is connected to the parallel bipolar electrode sensing platform, executing the most suitable driving voltages for the two light-emitting probes (CdTe QDs and g-C_3_N_4_ NSs) with their co-reactants. Applying a driving voltage of 9 V to the co-reactant K_2_S_2_O_8_ in the hydrophilic unit, which is in close contact with the cathode region of the parallel bipolar electrode, induces an excitation–radiative transition process with the emission of the light signal. Simultaneously, an oxidation reaction occurs at the anode when in contact with the solution containing H_2_O_2_, resulting in the bipolar electrode facilitating the electron transfer between the cathode and anode path.

### 5.6. Surface-Enhanced Raman Spectroscopy (SERS)

Surface-enhanced Raman spectroscopy (SERS) is an analytical technique that enhances the Raman scattering signal of molecules adsorbed on or near metallic nanostructures. SERS exploits the localized surface plasmon resonance (LSPR) phenomenon, where incident light excites collective oscillations of conduction electrons in metal nanostructures. This results in an enhancement of the Raman scattering signal by several orders of magnitude compared to conventional Raman spectroscopy. The enhancement arises from two mechanisms: electromagnetic enhancement due to the strong electromagnetic fields near the metal surface and chemical enhancement due to charge transfer between the molecule and the metal surface.

By incorporating SERS-active substrates onto paper substrates, researchers have created SERS-enhanced paper devices for on-site and point-of-care applications. The fabrication of SERS-active substrates involves the synthesis of noble metal nanoparticles (e.g., gold or silver) and their deposition onto paper substrates. Various methods, such as chemical reduction, physical deposition, inkjet printing, and lithography techniques, are employed to fabricate reproducible and uniform SERS substrates with high enhancement factors [124,125].

These devices have been used for the qualitative and quantitative analysis of various analytes, including chemicals, biomolecules, and pathogens. In healthcare, they can be used for the rapid and sensitive detection of biomarkers for disease diagnosis, monitoring of therapeutic drug levels, and detection of infectious agents. In environmental monitoring, SERS-based paper devices enable the detection of pollutants, toxins, and heavy metals in water, air, and soil. In food safety, they facilitate the identification of contaminants, adulterants, and allergens in food products.

## 6. Signal Readout Approach

### 6.1. Qualitative

Qualitative readout methods focus on determining the presence or absence of a particular analyte within a sample through visual inspection or colorimetric assays. Color is one of the most common signals in daily life, and a change in color can be easily distinguished by the naked eye. In the traditional colorimetric detection assay, color changes at the test zone depend on the concentration of the target (i.e., color intensity is proportional to analyte concentration). One common example is a paper-based point-of-care pregnancy kit with a colorimetric signal readout, which offers a convenient and accessible solution for the early detection of pregnancy. It operates on the principle of detecting human chorionic gonadotropin (hCG), a hormone produced during pregnancy, in urine samples. When hCG is present, it triggers a chemical reaction that produces a visible color change on the paper strip. This change serves as a positive indication of pregnancy. These types of paper-based devices provide YES or NO information (i.e., subjective interpretation) and are suitable for point-of-care diagnostics in resource-limited settings.

### 6.2. Quantitative

Quantitative analysis involves providing numerical data concerning the concentration or quantity of the target analyte in a sample. Meanwhile, sensing techniques such as fluorescence and electrochemical-based sensing offer quantitative signal readouts. As discussed previously, fluorescence-based paper sensors utilize fluorescent molecules that emit light of a specific wavelength upon excitation by an external light source. The presence of the target analyte induces a change in fluorescence intensity directly proportional to the analyte concentration. This alteration can be quantitatively assessed using a fluorescence reader or imaging system.

Furthermore, the results of a colorimetric assay, characterized by a visible color change, can also be quantified through digital image analysis using tools such as digital cameras and smartphones [126].

Quantitative analysis presents several notable advantages, including the precise and accurate quantification of analytes, detection of low concentrations of target molecules, and the ability to monitor dynamic changes in analyte levels. However, it necessitates complex instrumentation and accessories and may involve more intricate sample-preparation procedures.

## 7. Applications in Health Sensing

### 7.1. Diagnostic Assays for Infectious Diseases and Others Analytes

Paper-based point-of-care (POC) diagnostic devices have garnered significant attention due to their portability, cost-effectiveness, biodegradability, and ease of use [127]. These devices leverage the unique properties of paper substrates to perform various diagnostic assays, making them promising tools, especially in resource-limited settings, which fulfill the World Health Organization’s POC device development guidelines. Paper-based diagnostics typically involve using paper strips or cards that can wick biological samples, such as blood, saliva, or urine, through channels or zones containing reagents for specific assays [128].

**Figure 5:**
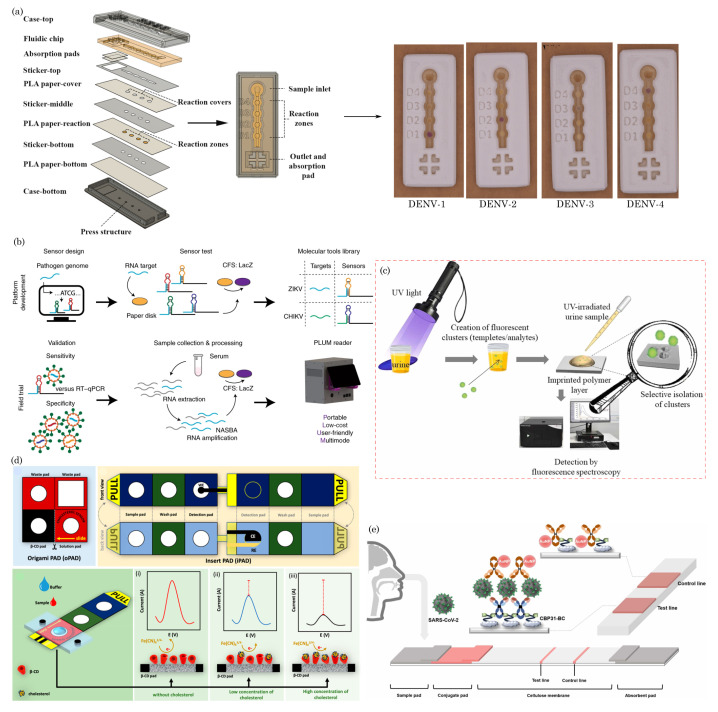
(**a**) Schematics of device assembly and photographs of visual color changes for different dengue virus serotypes. Reprinted with permission from Suvanasuthi et al. [136]. ©2021 Elsevier B.V. (**b**) Schematics of paper-based platforms for detecting the Zika and chikungunya viruses in serum samples. Reprinted with permission from Karlikow et al. [137]. Licensed under a Creative Commons Attribution 4.0 International License, ©2022 The Author(s). (**c**) Illustration of a paper-based analytical device for detecting prostate cancer using UV-irradiated urine samples. Reprinted with permission from Bezdekova et al. [153]. ©2023 Elsevier B.V. (**d**) The design concept of the 3D-ePAD, incorporating origami PAD (oPAD) and insert PAD (iPAD), and the detection mechanism for cholesterol across different concentrations. Reprint with permission from Chaiyo et al. [154]. Licensed under CC-BY-NC-ND 4.0. ©2024 The Authors. (**e**) Schematic of the CBP31-BC-based LFIA for detecting SARS-CoV-2. Reprinted with permission from Lee et al. [162]. ©2022 Elsevier B.V.

Paper-based microfluidic devices have been used for the point-of-care testing of vector-borne and flavivirus families such as malaria [129,130], dengue virus [131,132,133], and Zika virus [134,135]. For example, Suvanasuthi et al. [136] introduced a paper-based colorimetric biosensor for detecting dengue virus serotypes (DENV1-4). The paper substrate’s hydrophobic barriers were fabricated using 3D printing with polylactic acid (PLA) and wax filaments. The developed prototype demonstrated the ability to differentiate between dengue virus serotypes based on subtle nucleotide sequence variations. Figure 5a illustrates the schematics of device assembly and provides photographs showing the visual color changes corresponding to different dengue virus serotypes. Karlikow et al. [137] introduced a paper-based diagnostic platform for detecting Zika and chikungunya viruses in serum samples. The tests achieved high accuracy and sensitivity by utilizing a cell-free expression system, isothermal amplification, toehold-switch reactions, and a custom portable reader and computer vision-enabled image analysis software. Figure 5b depicts the detection mechanism schematics of the paper-based device. In suspected infection cases, the tests demonstrated accuracies of 98.5% for both Zika (95% confidence interval, 96.2–99.6%, 268 serum samples) and chikungunya (95% confidence interval, 91.7–100%, 65 serum samples) viruses, with sensitivities ranging from 2 aM to 5 fM, falling within clinically relevant concentrations. The prototype’s performance was successfully validated in field conditions.

Moreover, paper-based devices have been employed to diagnose other diseases and analytes, including influenza virus H5N1 [138], Neisseria meningitides [139], nucleic acid detection [140,141], noncommunicable diseases [142], cancer diagnosis [57,143,144], chronic obstructive pulmonary disease (COPD) biomarkers [145], HIV [146,147], pregnancy, infertility [148,149], and bioanalytes (uric acid, glucose, H_2_O_2_, and cholesterol) [150,151,152], etc. In a recent study, Bezdekova et al. [153] introduced a proof-of-concept paper-based device for diagnosing prostate cancer (CaP) from urine samples. Initially, urine samples underwent UV irradiation to induce the formation of fluorescent clusters. Subsequently, a selective molecularly imprinted polymeric layer was prepared on a paper substrate, allowing for the specific capture of these UV-induced fluorescent clusters within the urine sample to be diagnosed. Figure 5c illustrates the process of the formation, capture, and detection of CaP-specific clusters in UV-irradiated urine samples. These clusters, captured using molecular imprinting technology, are then quantified using fluorescence spectroscopy.

Chaiyo et al. [154] introduced a novel 3D electrochemical paper-based analytical device (3D-ePAD) coupled with near-field communication (NFC) potentiostat for the nonenzymatic detection of cholesterol. Figure 5d illustrates the design of the paper device and the strategies employed for cholesterol detection. This integrated platform comprises an origami PAD (oPAD) and an inset PAD (iPAD). β-Cyclodextrin (β-CD) immobilized on the oPAD is the specific material for cholesterol detection without enzymes. The device seamlessly integrates cholesterol detection with a battery-free NFC potentiostat on a smartphone. Cholesterol concentration is assessed through a [Fe(CN)_6_]^3−/4−^ current signal, a redox indicator stored in the detection section of the iPAD. The 3D-ePAD/NFC system demonstrates a linear detection range of 1–500 μM and a maximum detection limit of 0.3 μM for cholesterol. Furthermore, the sensor effectively measures cholesterol levels in real human serum samples, yielding results consistent with those obtained from a commercial cholesterol meter.

Most recently, during the COVID-19 pandemic, μPADs have played a pivotal role in point-of-care initial disease screening [155,156,157,158,159,160,161]. In one such example, Lee et al. [162] developed a colorimetric lateral flow immunoassay (LFIA) using a recombinant protein linker CBP31-BC to immobilize antibodies on a cellulose membrane in an oriented manner. Figure 5e shows the schematic of the CBP31-BC-based LFIA for detecting SARS-CoV-2. This LFIA demonstrated the sensitive detection of cultured SARS-CoV-2 in 15 min, with a low detection limit of $5\times 10^{4}$ copies/mL. Clinical evaluation using 19 samples validated by a reverse transcription–polymerase chain reaction (RT-PCR) revealed 100% accuracy in detecting positive and negative samples, even those with low viral loads.

These POC devices have been instrumental in enabling rapid and real-time testing for the SARS-CoV-2 virus, facilitating the early identification and containment of infections. Their ease of use, cost-effectiveness, and portability have made them particularly valuable in various settings, including clinics, airports, and resource-limited areas.

### 7.2. Wearable and Portable Health-Monitoring Devices

**Figure 6:**
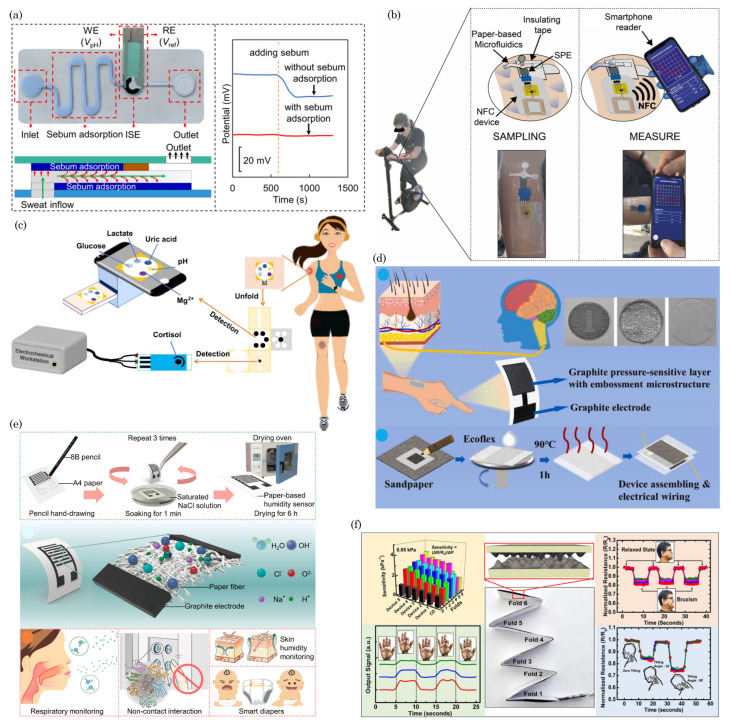
(**a**) Paper-based sandwich-structured sweat sensor with flow schematics and response diagram. Reprinted with permission from Yang et al. [171]. ©2023 American Chemical Society. (**b**) Cortisol monitoring during physical activity: Illustration of the sampling process, data measurement, and wireless transmission to a smartphone via NFC. Reprinted with permission from Fiore et al. [172]. ©2022 Elsevier B.V. (**c**) Schematics of wearable sweat sensor with an origami-based 3D paper structure for simultaneous analysis of multiple biomarkers (glucose, lactate, uric acid, magnesium ions, and pH value). Reprinted with permission from Cheng et al. [173]. Licensed under a Creative Commons Attribution 4.0 International License. (**d**) Biological and bioinspired tactile sensation system: Schematic of skin’s tactile function, a successful coin reproduction using the PGF method, and the graphite-based pressure-sensitive e-skin with embossed microstructure and electrodes. Fabrication process schematic of the PGF-based graphite e-skin. Reprinted with permission from Lai et al. [174]. ©2024 Elsevier B.V. (**e**) Schematics diagram depicting a pencil-on-paper hydration sensor designed to monitor physiological signals and characterize the skin barrier function. Reprinted with permission from Niu et al. [175]. ©2022 American Chemical Society. (**f**) Schematic diagram of the origami-inspired folded tactile sensor for human stimuli detection. Reprinted with permission from Karmakar et al. [176]. ©2023 American Chemical Society.

Wearable health-monitoring devices utilizing paper-based microfluidic technology represent a cutting-edge application at the intersection of healthcare and materials science. These devices offer a novel approach to continuous health monitoring, leveraging the unique properties of paper microfluidics to create flexible, lightweight, and cost-effective wearable sensors [163,164,165]. Paper-based biosensors have shown efficacy in detecting specific biomarkers associated with various health conditions. Wearable devices utilizing these biosensors offer the real-time monitoring of conditions such as diabetes, cardiovascular diseases, and infectious diseases, fostering a proactive approach to healthcare. Such wearable health monitors could measure parameters like biophysical features (body temperature, blood pressure, heart rate, and biopotential), sweat biochemicals (pH, uric acid, glucose, cholesterol, cortisol, etc.), lactate, or specific proteins, offering valuable data for individuals managing chronic conditions or athletes optimizing their performance [15,166,167,168,169,170]. For example, Yang et al. [171] designed a paper-based sandwich-structured wearable pH sensor with in situ sebum filtering for reduced interference (Figure 6a). The sensor comprised five layers: a PDMS-based cover layer, a sebum adsorption or ISE top layer (microfluidic snake channel), a filter-paper-based middle microfluidic layer, a sebum adsorption bottom layer (microfluidic snake channel), and an adhesive layer made of double-sided medical adhesive tape for skin fixation. Sweat, introduced through the inlet, moved along the microfluidic layer, allowing sebum adsorption. The sensor effectively adsorbed sebum mixed in sweat, ensuring an accurate pH measurement and facilitating sweat evaporation through the outlet window. Fiore et al. [172] innovated paper-based electrochemical biosensors for cortisol detection in sweat, a stress biomarker (see scheme, Figure 6b). The device uses filter paper for a reagent-free, competitive magnetic-bead-based immunosensor to orchestrate flow and reagent loading. Fabricated with filter paper and solid wax-based printing, the microfluidic pattern features hydrophilic channels defined by hydrophobic wax barriers. Magnetic beads, functionalized with monoclonal antibodies, facilitate specific cortisol measurement in the reaction zone. Integration with a near-field communication wireless module yields a flexible, wearable analytical tool for cortisol detection in sweat. Cheng et al. [173] devised a 3D origami-based μPADs wearable biosensor for multiplexed analyte detection in sweat. Figure 6c illustrates the schematic of a wearable sweat sensor featuring an origami-based 3D paper structure designed for the simultaneous analysis of multiple biomarkers. The square-shaped wearable sweat chip, measuring 36 mm on each side, featured a microfluidic channel with distinct layers for effective analysis. The 3D channel incorporated a collection layer, vertical and horizontal channels, an electrode layer, a colorimetric sensing layer, and a sweat evaporation layer. Screen-printed electrodes were employed for cortisol measurement, while the colorimetric sensing layer utilized cotton-thread-based channels. Sweat absorbed through the collection layer underwent a chromatographic process, reacting at the electrode layer and flowing into the lateral channel for colorimetric analysis. The chip enabled electrochemical and colorimetric sensing, with image analysis conducted using ImageJ and the electrochemical workstation. Recently, Lai et al. [174] presented an ultralight and highly sensitive biological and bioinspired tactile sensation system using printing paper to monitor human wrist pulses, acoustical vibration, and information encryption. The skin’s schematic fabrication involves pencil graphite frottage (PGF) for the pressure-sensitive film, creating extended graphite electrodes through pencil writing, and eliminating metal electrodes from the process. A protective ecoflex film is spin-coated onto the printing paper’s back, providing a self-adhesive layer. The final e-skin is assembled by placing two graphite-coated printing papers facing each other, with copper wires attached to the graphite electrodes. This innovative approach achieves a versatile and lightweight tactile sensing system. Figure 6d shows a schematic of the skin’s tactile function transmitting action potentials to the brain via nerves. In the top right, the photograph shows the successful reproduction of a coin design using the PGF method. At the bottom is the schematic of a graphite-based pressure-sensitive e-skin structure for tactile sensing, comprising a graphite pressure-sensitive layer with an embossed microstructure and graphite electrodes. Niu et al. [175] introduced a pencil-made paper-based hydration sensor for health monitoring, particularly respiratory monitoring, noncontact switching, and skin characterizations. Figure 6e illustrates the fabrication process and response mechanism of the flexible pencil-on-paper hydration sensor, showcasing its potential applications in health monitoring, noncontact switching, and skin characterization. The design and fabrication approaches proposed in this study offer opportunities for the future development of wearable, self-powered, and recyclable sensors and actuators. Karmakar et al. [176] pioneered the development of an origami-inspired conductive paper-based folded pressure sensor tailored for detecting human stimuli. In Figure 6f, the sensor schematics and sensing mechanisms are illustrated, showcasing the intricate design and functionality of the sensor. This innovative sensor design draws inspiration from the principles of origami, leveraging folding techniques to create a flexible and responsive sensor capable of detecting various stimuli. The intricate folding patterns and conductive materials integrated into the paper-based sensor enable the precise detection and measurement of pressure changes, making it suitable for applications in human–computer interaction, wearable technology, and biomedical sensing.

### 7.3. Animal Health Screening

Animal health is critical in various sectors, including agriculture, veterinary medicine, and food production. The timely and accurate screening of animal health parameters is essential for disease diagnosis, surveillance, and control. Conventional methods for animal health screening often involve complex and time-consuming laboratory procedures, which may not be suitable for on-site or point-of-care testing. Paper-based microfluidics has emerged as a promising technology that offers a promising alternative due to its portability; low cost; and ability to perform the rapid, sensitive, and specific detection of various analytes.

**Figure 7:**
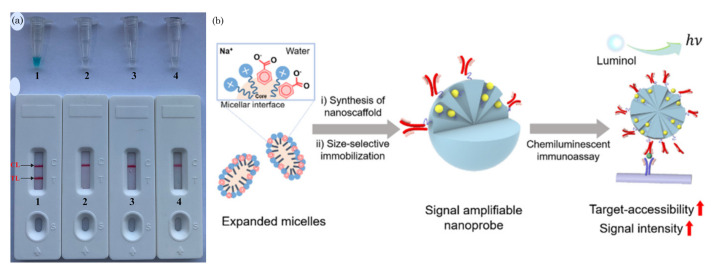
(**a**) Photographs showing the confirmation and verification of *Brucella*-MCDA products: color change in *Brucella*-MCDA tubes (**top**) and visual detection through LFAs (**bottom**) of the presence of *Brucella* species. Tube 1 (biosensor 1): positive amplification; tube 2 (biosensor 2): negative amplification (*Salmonella*), tube 3 (biosensor 3): negative amplification (*Bacillus cereus*), tube 4 (biosensor 4): negative control (DW). Reprinted with permission from [182]. ©2019 The Authors. This open-access article is distributed under the terms of the Creative Commons Attribution License (CC BY). (**b**) Scheme for detection of avian influenza viruses (AIVs) using a signal-amplifiable nanoprobe-based chemiluminescent lateral flow immunoassay (CL-LFA), reprinted with permission from Jung et al. [183], ©2020 American Chemical Society.

Several studies have demonstrated the utility of paper-based microfluidics devices in animal health screening, including disease diagnosis, monitoring of biomarkers, and detecting pathogens [177,178,179,180,181]. For example, research by Li et al. [182] demonstrated the utility of paper-based lateral flow biosensors (LFB) for the highly specific, simple, rapid, and visual detection of *Brucella*-specific amplicons (See Figure 7a). Their device utilized *Brucella*-MCDA-functionalized paper strips to capture and detect the *Bscp31* gene (*Brucella* species-specific gene), offering a rapid and cost-effective method for on-site screening. Similarly, Jung et al. [183] showcased the development of a signal-amplifiable nanoprobe-based chemiluminescent lateral flow immunoassay (CL-LFA) for the detection of avian influenza viruses (AIVs) and other viral avian-origin diseases, offering a low-cost alternative to conventional diagnostic methods (See Figure 7b). The nanoprobe allows for the selective immobilization of antibodies and enzymes on sensitive paper-based sensor platforms, enabling enhanced detection sensitivity. Tests conducted with low pathogenicity avian influenza H9N2, H1N1, and high pathogenicity avian influenza H5N9 viruses showed detection limits of 10^3.5^ to 10^4^ 50% egg infective dose (EID_50_)/mL, significantly lower than those of commercial AIV rapid test kits. The CL-LFA also demonstrated high sensitivity and specificity against clinical samples, indicating its potential as a diagnostic tool for sensitive antigen detection in clinical settings.

## 8. Environmental Monitoring/Sensing

Environmental pollutants, spanning heavy metals, organic compounds, pathogens, airborne pollutants, and other hazardous substances, pose severe threats to ecosystems and human health [184,185,186]. Persistent soil and water contamination with heavy metals such as lead, mercury, cadmium, and arsenic is primarily attributed to industrial activities, mining, and improper waste disposal. Contributing to organic pollution in water bodies, organic compounds like pesticides, herbicides, industrial chemicals, and pharmaceuticals impact aquatic life and may enter the human food chain. Waterborne pathogens, encompassing bacteria, viruses, and microorganisms, pose health risks, necessitating effective monitoring systems. Additionally, airborne pollutants, such as particulate matter, volatile organic compounds (VOCs), nitrogen dioxide, and sulfur dioxide, contribute to air pollution, impacting respiratory health and disrupting ecosystem balance. Addressing these multifaceted challenges requires comprehensive monitoring and mitigation strategies.

### 8.1. Detection of Soil Contaminants

Soil contaminants significantly threaten environmental ecosystems and human health, necessitating efficient early detection and mitigation monitoring systems. Paper-based microfluidic devices have emerged as promising tools in soil contaminant monitoring due to their cost-effectiveness, simplicity, and portability. These devices leverage the capillary action of paper to facilitate the flow of liquids through microchannels, allowing for the detection of various contaminants. They are well-suited for applications in resource-limited settings where sophisticated laboratory equipment may be impractical. These paper-based systems can be designed to detect a range of soil contaminants, including heavy metals, pesticides, and organic pollutants.

**Figure 8:**
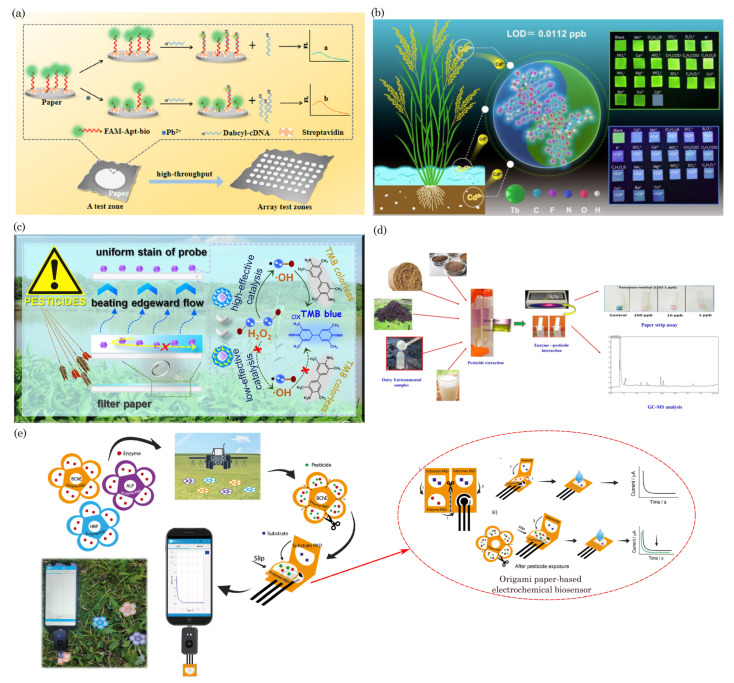
(**a**) Illustration of a high-throughput paper-based FRET aptasensor designed for the detection of Pb^2+^. Reprinted with permission from Suo et al. [187]. ©2022 Elsevier B.V. (**b**) Schematics of fiber-made filter-paper-based biosensors for the real-time monitoring of Cd^2+^ in water, rice, and rice soil. Reprinted with permission from Yu et al. [188]. ©2023 American Chemical Society. (**c**) Principle illustration of the paper-based colorimetric sensor for real-time monitoring of pesticides. Reprinted with permission from Zhang et al. [193] ©2022 Elsevier B.V. (**d**) Schematic illustration of pesticide detection in dairy samples using the paper-based sensor. Reprinted from Ranveer et al. [194] under a Creative Commons license ©2022 Elsevier B.V. (**e**) Schematic representation of the origami paper-based electrochemical biosensor for pesticide detection. Reprinted with permission from Caratelli et al. [195]. ©2022 Elsevier B.V.

Suo et al. [187] developed a high-throughput paper-based fluorescence resonance energy transfer (FRET) aptasensor for the sensitive detection of low concentrations of Pb^2+^. Figure 8a illustrates the detection methodology. Fabricated on Whatman No. 1 chromatographic paper, the device demonstrated the capability to detect Pb^2+^ in a concentration range spanning from 0.01 to 10 μM, with an impressive limit of detection (LOD) of 6.1 nM. This innovative strategy successfully analyzed various real samples, including water, soil, and food, showcasing its applicability in practical scenarios for environmental and food safety assessments. Integrating FRET technology into a paper-based platform enhances the efficiency and throughput of the aptasensor, offering a versatile and sensitive tool for rapid detection in diverse sample matrices. Yu et al. [188] introduced a fiber-made filter-paper-based device for the real-time monitoring of Cd^2+^ in water, rice, and rice soil (see Figure 8b). The developed test paper exhibited highly sensitive and visible sensing capabilities for Cd^2+^ in water, rice supernatants, and rice soil supernatants. The LODs in these real samples were remarkably low, measuring at 0.0112 ppb for water, 1.1240 ppb for rice supernatants, and 0.1124 ppb for rice soil supernatants. These LODs were found to be lower than the national standards (GB 2762-2022) for food safety in China [189], underscoring the device’s potential for precise and reliable detection, with implications for ensuring compliance with stringent safety regulations in diverse environmental and agricultural settings.

Furthermore, pesticides, vital for ensuring food security by controlling pests, weeds, and plant diseases, have significantly increased food availability over the past 50 years. However, their widespread use has led to environmental pollution, adversely affecting ecosystems and human health [190,191]. Implementing efficient management practices and user-friendly point-source monitoring systems accessible to farmers can alleviate pesticides’ environmental and health impacts. In this context, paper-microfluidics-based devices have played a significant role in pesticide detection [192]. Zhang et al. [193] developed an innovative paper-based colorimetric sensor for thiacloprid, a commonly used agricultural pesticide, with a low detection limit of 0.04 μM. Figure 8c shows the schematic representation of the principle behind the paper-based colorimetric sensor designed for the real-time monitoring of pesticides. The quantification of the sensor’s output was facilitated through RGB analysis, providing a simple and efficient method for detection. Notably, integrating a smartphone app for output reading enhances the accessibility and user-friendliness of the paper-based sensor, offering a promising solution for on-site and real-time monitoring of thiacloprid levels in agricultural settings. Ranveer et al. [194] designed a versatile paper-based dipstick assay for the colorimetric detection of fungicides, organochlorines, organophosphates, carbamates, and herbicides in diverse matrices such as animal feed, water, milk, and soil. Figure 8d presents a schematic illustration depicting the detection of pesticides in dairy samples through the paper-based sensor. The developed dipstick demonstrated versatile applicability with an impressive LOD for different pesticide groups. Specifically, the LOD values ranged from 1 to 10 μg L^−1^ for fungicides, 1 to 50 μg L^−1^ for organochlorines, 250 to 500 μg L^−1^ for organophosphates, 1 to 50 μg L^−1^ for carbamates, and 1 μg L^−1^ for herbicides. This paper-based assay showcased sensitivity across a range of pesticide residues. It illustrated its potential as a rapid and cost-effective tool for assessing pesticide contamination in multiple environmental and food matrices. Caratelli et al. [195] introduced a 3D flower-like origami paper-based device designed for the electrochemical detection of pesticides, specifically paraoxon, 2,4-dichloro phenoxy acetic acid, and glyphosate, in the aerosol phase, catering to applications in precision agriculture. Figure 8e illustrates a schematic representation of the electrochemical biosensor for pesticide detection based on an origami-based paper device. The innovative device was seamlessly integrated with a smartphone for convenient output reading. Remarkably, this paper-based system demonstrated the efficient detection of the three classes of pesticides in the aerosol phase, achieving impressive LODs equal to 30 ppb, 10 ppb, and 2 ppb for 2,4-D, glyphosate, and paraoxon, respectively. Integrating electrochemical sensing with a portable paper-based platform enhances accessibility and usability, offering a promising tool for real-time pesticide monitoring in agricultural settings with potential implications for sustainable and precise farming practices.

Paper-based sensors have been found to have a noteworthy application in detecting explosive residues in soil, presenting a valuable forensic-oriented environmental monitoring and security tool [196,197,198,199,200]. The unique attributes of paper microfluidic devices, such as their portability, simplicity, and cost-effectiveness, make them well-suited for the on-site detection of explosive remnants. These sensors can be tailored to detect specific volatile compounds, offering a targeted and efficient approach to soil analysis. The detection mechanism often involves incorporating reactive agents or biomolecules onto the paper substrate, allowing for a rapid and selective response to the presence of explosive residues. This application is particularly crucial in areas where the remnants of explosives pose environmental and safety concerns, such as former military sites or regions affected by conflict. By leveraging the capabilities of paper-based sensors, ecological professionals and security personnel can conduct real-time, on-site assessments of soil contamination, facilitating prompt remediation efforts and contributing to a safer and more secure environment.

### 8.2. Water Quality Monitoring

Water quality monitoring ensures clean and safe drinking water access, addressing public health and ecological concerns [201]. Unfortunately, many developing regions face challenges meeting this fundamental need due to inadequate water treatment plans and infrastructure. Countries are grappling with water quality issues due to the rapid growth of human activities like urbanization and industrialization, leading to significant pollution [202]. Thus, ensuring access to clean water has emerged as a significant challenge in recent decades, impacting developing and developed nations. Traditional methods for detecting water contaminants involve chromatographic and spectroscopic techniques, necessitating costly equipment and specialized personnel [203]. Despite the emergence of water toxicity biosensors employing enzymes, antibodies, and microorganisms in recent decades, their specificity limits them to known chemicals, rendering them unsuitable for monitoring unforeseen contaminants in water [204,205]. Addressing the pressing need for on-site and real-time measurements of toxic components in water, there is a demand for a rapid and portable sensor.

**Figure 9:**
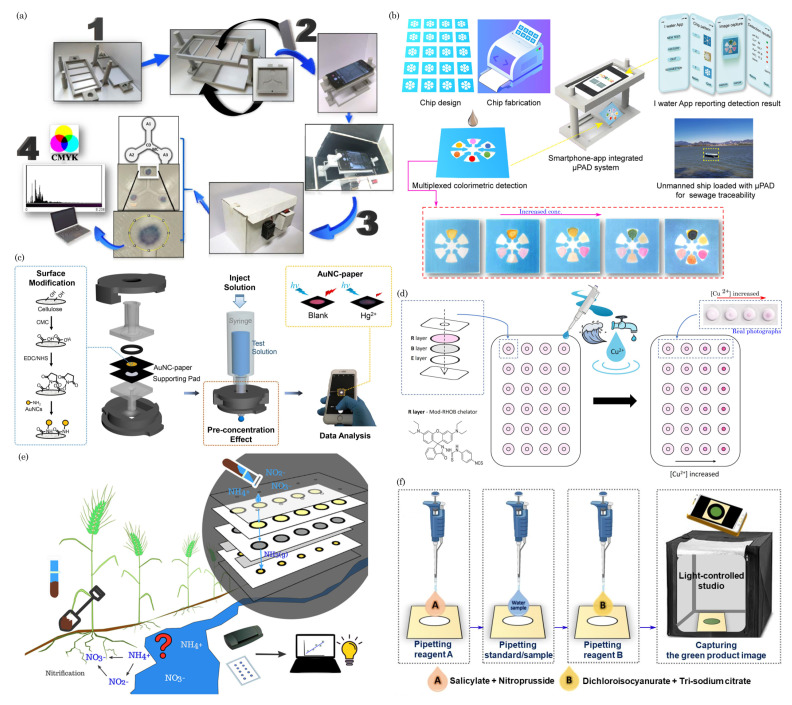
(**a**) Illustration of the μPAD assembly: production of smartphone support parts, coupling smartphone support with 3D-printed μPAD support, assembly of the “closed box” with integrated support and LED white light, and data acquisition through smartphone image capture and conversion of RGB to CMYK color standards using ImageJ® software. Reprinted with permission from Da Silva et al. [212]. ©2020 Elsevier Ltd. (**b**) The schematic diagram illustrates the fabrication process of the μPAD, the multiplexed colorimetric detection strategies, and the integration of a smartphone app for its applications. Reprinted with permission from Xiong et al. [213]. ©2022 The Authors, published by American Chemical Society, licensed under CC BY-NC-ND 4.0. (**c**) Schematic illustration of the gold nanoclusters (AuNC)-modified paper device designed for detecting Hg^2+^ ions. Reprinted with permission from Lin et al. [214]. ©2021 Elsevier B.V. (**d**) Illustration of the μPAD assembly designed for copper determination in water, along with actual photographs of the paper device depicting varying concentrations of Cu^2+^. The components include laminating pouch sheets L1 and L2, a reagent layer R, a buffer layer B, and an empty layer E. Reproduced with permission from Aguiar et al. [215]. Under a Creative Commons license, ©2024 The Authors. Published by Elsevier B.V. (**e**) Schematic representation of the μPAD designed for detecting inorganic nitrogen in water and soil samples. Reprinted with permission from Uhlikova et al. [216]. Under a Creative Commons license, ©2024 The Authors. Published by Elsevier B.V. (**f**) Diagram depicting the analytical steps for the straightforward colorimetric determination of NH_4_^+^ utilizing the proposed paper-based analytical device. Reprinted with permission from Thangjitsirisin et al. [217]. ©2024 Elsevier B.V.

Paper-microfluidics-based devices have been instrumental in water quality monitoring in the past decades due to their versatility, cost-effectiveness, and ease of use [206,207,208,209,210,211]. For example, Da Silva et al. [212] presented an innovative approach by developing a μPAD tailored for the point-of-use colorimetric monitoring of water hardness, phenols, and pH. The fabrication process involved using a cutter printer and 3D printing to create these paper devices. The reading output of the device, specifically the discernible change in visible colors in the presence of analytes, was quantified through captured images using an integrated smartphone (Figure 9a). In a study by Xiong et al. [213], a colorimetric-based μPAD was developed for the simultaneous detection of diverse water quality parameters, including Cu(II), Ni(II), Fe(III), nitrite, and pH. The fabrication involved creating hydrophobic flow patterns on a Whatman Grade 1 filter paper substrate using a wax printer. The visible color changes on the paper device induced by the presence of Cu(II), Ni(II), Fe(III), nitrite, and pH were quantified through RGB analysis using a smartphone app. Figure 9b shows the schematics of the device assembly and detection methods. The device demonstrated impressive detection limits of 0.4 ppm for nitrite, 1.9 ppm for Cu(II), 2.9 ppm for Ni(II), 2.9 ppm for Fe(III), and 5 for pH, with rapid detection achieved within 5 min.

Lin et al. [214] developed a portable paper analytical device modified with nanoclusters and integrated with a syringe for highly sensitive Hg^2+^ detection. The device comprises a paper substrate modified with fluorescent gold nanoclusters (AuNC-paper) enclosed in a reusable cartridge connected to a syringe, facilitating the flow of a large sample volume through the paper for enhanced analyte signal accumulation. The schematic illustration in Figure 9c depicts the AuNC-modified paper device designed for Hg^2+^ ion detection. In the presence of Hg^2+^ ions, the color of the paper substrate changes visibly, enabling naked-eye detection. This technique allows Hg^2+^ ion detection within 30 min, achieving a low detection limit of 1.2 nM.

Aguiar et al. [215] recently presented a μPAD designed for copper detection in natural waters. The μPAD assembly comprises three filter paper discs (R: Whatman 42, B: Whatman 1, E: Whatman 3) with a 9.5 mm diameter, arranged in twenty-four hydrophilic units in each layer (Figure 9d). The R-layer paper discs were prepared by applying 12 μL of Mod-RHOB ligand solution to each disc and were oven-dried at 50 °C for 10 min. The B-layer paper discs were prepared with 10 μL of buffer solution and underwent the same drying process. The E-layer was left untreated. To determine the copper concentration, 20 μL of standard/sample was loaded onto the assembled μPAD through the sample holes, absorbing in approximately 2 min. The reaction between Mod-RHOB and copper produces a pink color complex in the R layer, intensifying with increasing copper concentration.

Uhlikova et al. [216] introduced a μPAD for the colorimetric detection of inorganic nitrogen in water and soil samples. Figure 9e illustrates the detection strategy employed by the device. The developed device demonstrated the capability to detect ammonium and nitrate using bromothymol blue (an acid–base indicator) with quantification limits of 6.5 and 18.2 mg N L^−1^, respectively. Similarly, using nitrazine yellow (another acid–base indicator), the quantification limits were found to be 2.1 and 4.2 mg N L^−1^ for ammonium and nitrate, respectively. The newly developed μPAD exhibited stability for 62 days when stored in a freezer and 1 day at ambient temperature. Validation with certified reference material confirmed its accuracy, and successful application was demonstrated in determining ammonium and nitrate in spiked environmental water samples and soil extracts.

More recently, Thangjitsirisin et al. [217] introduced a μPAD for the colorimetric determination of ammonium ions in water. The device utilized a superhydrophobic eggshell, an environmentally friendly material, to create a hydrophobic barrier on a circular Whatman No. 1 filter paper substrate. As depicted in Figure 9f, the yellowish color zone on the paper device indicates the presence of the hydrophobic ’eggshell’ barrier surrounding a hydrophilic reservoir. The figure also outlines the step-by-step method for the colorimetric detection of ammonium ions in water. The procedure involves pipetting a 3.0 μL aliquot of reagent A (salicylate and nitroprusside) onto the hydrophilic reservoir, followed by the transfer of a 3.0 μL aliquot of the water sample or a series of standard NH_4_^+^ solutions (5–100 mg N L^−1^). Subsequently, a 3.0 μL aliquot of reagent B (dichloroisocyanurate and tri-sodium citrate) is added, and after a 5 min reaction period, a visible color change occurs in the hydrophilic reservoir area. The device is then placed in a constant-light illumination studio for image capture and the quantification of color intensity.

Overall, these μPADs offer a promising solution for the on-site and simultaneous monitoring of multiple water quality parameters, showcasing its potential for environmental monitoring and water analysis applications.

### 8.3. Air Quality Monitoring/Gas Sensing

Air pollution poses a significant threat to public health and the environment. Conventional air quality monitoring systems are characterized by their high cost, limited portability, and dependency on sophisticated infrastructure. The emergence of paper-based microsystems presents a cost-effective and portable alternative, leveraging the inherent properties of paper substrates for the efficient detection of air pollutants [218,219,220].

**Figure 10:**
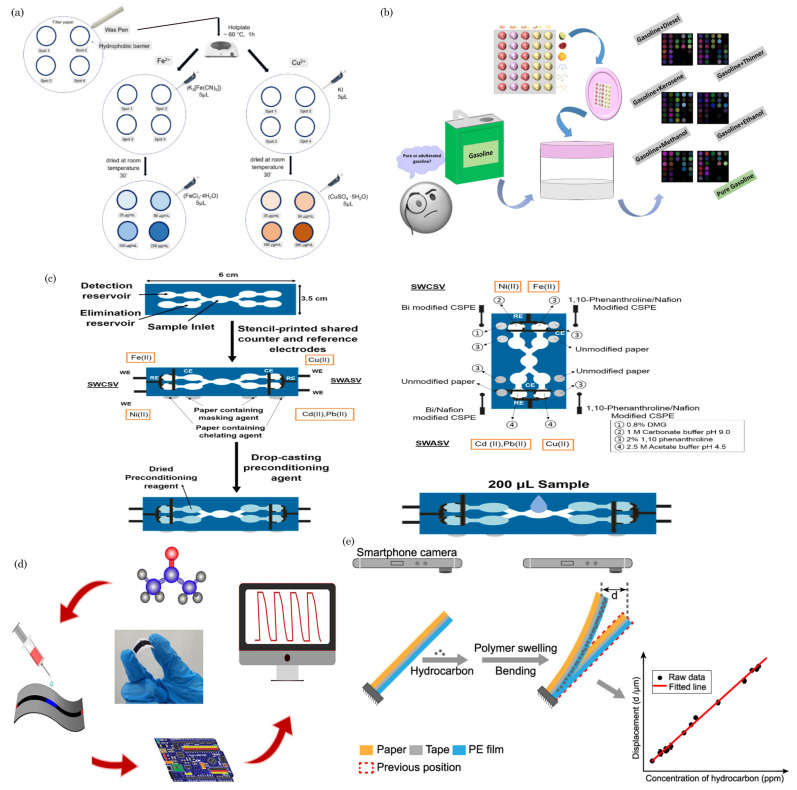
(**a**) Colorimetric detection of Fe^2+^, Cu^2+^ ions, reprinted with permission from De Matteis et al. [221] under Creative Commons Attribution (CC BY) license. ©2020 The authors, published by MDPI. (**b**) Schematic representation of colorimetric detection process of gasoline utilizing a paper-based optical nose. Reprinted with permission from Bordbar et al. [222]. ©2022 American Chemical Society. (**c**) Electrochemical detection of metals in aerosol samples using paper-based analytical device. Reprinted with permission from Mettakoonpitak et al. [225]. ©2019, American Chemical Society. (**d**) Schematics of the paper-based flexible sensors for detection of acetone at room temperature. Reprinted with permission from Davis et al. [226]. ©2023 American Chemical Society. (**e**) Sensing mechanism of the milli-cantilever. Reprinted with permission from Qin et al. [227]. ©2020 American Chemical Society.

Colorimetric paper-based sensors offer an innovative approach to environmental monitoring, especially in detecting air pollutants. In their work, De Matteis et al. [221] designed a paper-based analytical device (PAD) capable of detecting contaminants such as Fe^2+^, Cu^2+^ ions in water, and NH_3_ and C_2_H_4_O in the air, even at low concentrations. The researchers employed a wax pen to form a circular hydrophobic barrier on a Whatman filter paper substrate to create distinct sensing zones. These marked spots were utilized to detect the specified analytes at various concentrations. Figure 10a shows the schematics of the detection mechanisms. Notably, the paper sensor displayed a colorimetric response directly correlated with the concentration of the identified pollutant species.

Bordbar et al. [222] developed a paper-based optical nose by depositing bimetallic silver and gold nanoparticles onto a paper substrate, synthesized using both natural and chemical reducing agents. This assay was evaluated for its capability to distinguish between gasoline and five ignitable liquids: diesel, ethanol, methanol, kerosene, and thinner. The interaction between the sensor and sample vapors led to nanoparticle aggregation, resulting in color changes captured by a scanner, producing distinct colorimetric maps for each analyte (Figure 10b). Visual observations were corroborated using multivariate statistical analyses, including principal component analysis and hierarchical clustering analysis. Additionally, partial least-squares regression aided in estimating the quantities of ignitable liquids present as counterfeit substances in gasoline samples, with root mean square errors for prediction ranging from 1.7% to 3.4%. Ultimately, the fabricated sensor demonstrated high efficiency for the onsite detection of pure industrial gasoline samples versus adulterated ones.

Moreover, paper-based devices are extensively utilized for the electrochemical-based detection of air pollutants [223,224]. Mettakoonpitak et al. [225] introduced an innovative electrochemical paper-based device (ePAD) for the multiplexed detection of metals, specifically Cd, Pb, Cu, Fe, and Ni, from a single particulate matter sample. The paper-based device was designed with four independent channels and working electrodes, enabling the implementation of square-wave anodic stripping voltammetry (SWASV) and square-wave cathodic stripping voltammetry (SWCSV) for the simultaneous determination of multiple metals. Figure 10c shows an example of electrochemical-based paper sensors for air pollutant detection. Notably, the device exhibited impressive detection limits, ranging from 0.5 to 400.0 μg L^−1^ for Cd(II), Pb(II), and Fe(II); 1.0 to 400.0 μg L^−1^ for Cu(II); and 0.5 to 200.0 μg L^−1^ for Ni(II). This multiplexed ePAD offers a versatile and efficient solution for sensitively detecting various metals in complex samples, showcasing its potential for environmental monitoring and analytical applications.

Davis et al. [226] engineered a flexible paper-based sensor for acetone detection at room temperature. The paper-based electrodes were crafted through the application of zinc oxide (ZnO)-polyaniline-based conductive inks (Figure 10d). These electrodes exhibited remarkable conductivity (80 S/m) and stability under rigorous mechanical and chemical conditions while demonstrating commendable flexibility (1000 bending cycles). The acetone sensor displayed a notable sensitivity of 0.02/100 ppm and 0.6/10 μL, with a broad sensitivity range spanning from 260 to >1000 ppm under atmospheric conditions. Moreover, the sensors exhibited an impressive response time of 4 seconds and a recovery time of 15 s for acetone detection at room temperature without necessitating external heaters. The proposed paper device’s high sensitivity and long-term stability make it suitable for wearable biosensor applications.

Moreover, a cantilever-based paper-based sensor device was demonstrated by Qin et al. [227]. They developed an inexpensive and lightweight hydrocarbon gas sensor utilizing a smartphone camera for readout. The sensor relies on paper-based milli-cantilever bending induced by polymer swelling. The sensing cantilever comprises three layers: a functional layer of polyethylene film, an adhesive layer of double-sided tape, and a weighing paper substrate. Figure 10e shows schematics of the milli-cantilever. The milli-fabricated sensing cantilever has dimensions of 8 mm length, 0.5 mm width, and 50 μm thickness. The sensor’s response is measured as the displacement of the milli-cantilever-free end. Demonstrating its capabilities, the sensor exhibited a linear response to hydrocarbon concentrations, a broad detection range, low detection limits, and rapid response times. For instance, when exposed to xylene, the sensor displayed a detection range of 15–140 ppm, a low detection limit of 15 ppm, and a fast response time of 30 s.

## 9. Food Safety

Paper-based devices have emerged as valuable tools in ensuring food safety due to their simplicity, cost-effectiveness, and ease of use. These devices are designed to detect various contaminants and ensure the quality of food products. Here are some examples of paper-based devices for food safety applications.

**Figure 11:**
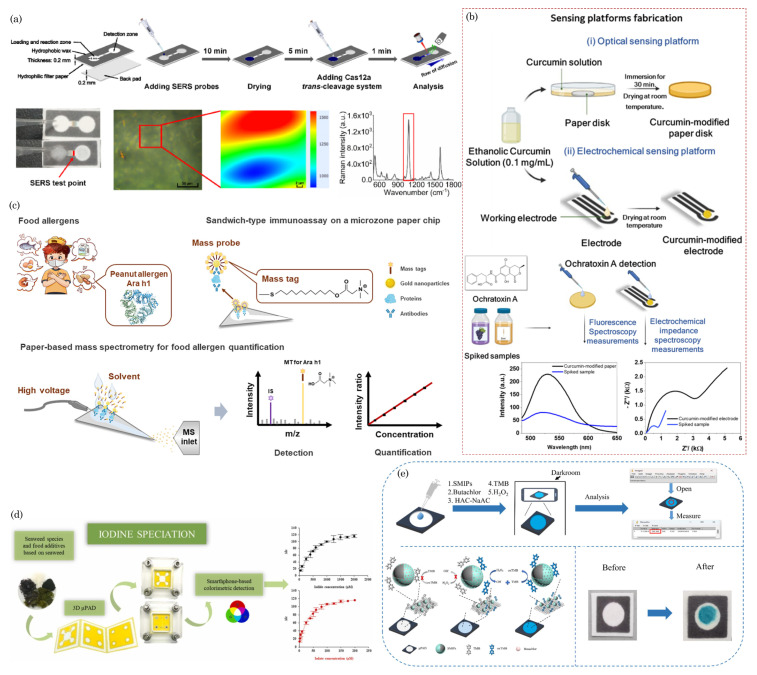
(**a**) Illustration of the device schematics, operational steps, and a microscopic image of the S. typhi test zone, showcasing SERS mapping signals at 1075 cm^−1^, alongside the corresponding Raman spectrum. Reprinted with permission from Zhuang et al. [228]. ©2022 Elsevier B.V. (**b**) Schematic representation of optical and electrochemical sensing platforms utilizing curcumin-immobilized paper substrates for ochratoxin A detection in grape juice and beer. Reprinted with permission from Dos Santos et al. [232] under a Creative Commons license, ©2023 The Author(s). Published by Elsevier B.V. (**c**) Schematic illustration of microzone paper-based mass spectrometric immunoassay for detecting food allergens (peanut allergen Ara h1). Reprinted with permission from Lu et al. [239]. ©2024 American Chemical Society. (**d**) Schematics of 3D μPAD with colorimetric detection for iodine speciation in seaweed samples. Reprinted with permission from Placer et al. [242] under a Creative Commons license, ©2022 The Authors. Published by Elsevier B.V. (**e**) The procedural steps of SMIPs-μPAD and actual images of the paper device before and after color development. Reprinted with permission from Wu et al. [248]. ©2023 Elsevier Ltd.

Paper-based devices are widely used to rapidly detect foodborne pathogens such as *Salmonella*, *E. coli*, and *Listeria*. These devices often employ antibodies or DNA probes to capture and identify specific pathogens, providing quick results for on-site testing. For example, Zhuang et al. [228] developed an integrated microfluidic paper-based analytical device, termed RPA-Cas12a-μPAD, combining recombinase polymerase amplification (RPA) with supersensitive surface-enhanced Raman scattering (SERS) detection. Figure 11a illustrates the device schematics, operational steps, and microscopic image of the S. typhi test zone with SERS mapping signals at 1075 cm^−1^, along with the corresponding Raman spectrum. The successful detection of *Salmonella* in milk and meat samples was achieved with detection limits of 3.72 and 4.04 CFU/mL, respectively.

The detection of toxins in food, such as mycotoxins and chemical contaminants, is critical for ensuring food safety. Paper-based assays can detect specific toxins through colorimetric or electrochemical reactions, providing a visual indication of contamination [229,230,231]. Dos Santos et al. [232] developed curcumin-modified paper-based sensing platforms for detecting ochratoxin A (OTA) in grape juice and beer samples (Figure 11b). The sensor operates based on specific interactions between curcumin and OTA, involving energy and electron transfer mechanisms in optical detection. Curcumin molecules form complexes with OTA in electrochemical detection, enhancing the binding affinity between OTA and the electrode surface. This results in a greater change in the impedance of the double layer, easily detected by electrochemical impedance spectroscopy (EIS). Sensors exhibit good sensitivity, with limits of detection (LODs) of 0.09 ng/mL and 0.045 ng/mL for optical and electrochemical methods, respectively, remaining effective across various food matrices and in the presence of potential interferents.

Paper-based tests are employed to detect allergens, helping to prevent allergic reactions in individuals with specific sensitivities. These devices can detect the presence of allergenic proteins, allowing for rapid screening in various food products such as ovalbumin and egg white protein [233], milk allergen (β-lactoglobulin) [234,235,236], histamine in canned tuna [237], and peanut allergen Ara h1 [238]. Recently, Lu et al. [239] developed a paper-based mass spectrometric immunoassay platform for peanut allergen detection. Figure 11c illustrates the microzone paper-based mass spectrometric immunoassay for food allergen detection schematic. They introduced a novel quaternary ammonium-based mass tag and a paper chip with a microzone, resulting in significant signal enhancement. This method could detect Ara h1 with a linear range of 0.1–100 ng/mL and a detection limit of 0.08 ng/mL in milk matrices. Moreover, it accurately quantified Ara h1 in various milk-related beverages, biscuits, and candy bars with complex matrices, demonstrating a capability for low-concentration quantitation.

Paper-based devices are utilized to assess the quality of food products. For example, pH strips on paper can indicate a product’s acidity (e.g., carbendazim detection on the skin of apple and cabbage [240] and acidic pH and bisulfite in white wine [241]), ensuring it meets quality standards. Similarly, these devices can monitor the freshness of certain perishable items. Another example is the detection of iodine speciation in seaweed samples. Placer et al. [242] engineered a 3D origami microfluidic paper-based analytical device for quantifying iodide and iodate levels in edible seaweeds via smartphone-based colorimetric detection. The paper device was predesigned to generate hydrophobic patterns on Whatman No. 1 filter paper using wax printing and assembled by folding the paper substrates. Figure 11d illustrates the fabrication of the detection device and plots the analytical signal against analyte concentrations.

The detection of food adulterants and contaminants, such as pesticides or additives, is crucial for maintaining food safety. Paper-based assays can be tailored to identify specific adulterants through selective reactions, quickly assessing food purity. Recent examples include the detection of adulteration in Iranian honey [243] and milk adulteration with melamine [244], starch [245], sugar [246], and urea [247]. Wu et al. [248] developed a surface molecularly imprinted microfluidic paper-based device (SMIPs-μPAD) for detecting butachlor in mung bean samples. When combined with a smartphone, this colorimetric paper chip demonstrated high selectivity and sensitivity to butachlor, with a detection limit of 1.43 ng/g and a detection time of 20 min. Figure 11e depicts the operation steps of SMIPs-μPAD and real photographs of the paper device before and after color development.

## 10. Biodegradability and Sustainability

### 10.1. Environmental Impact of Traditional Microfluidic Devices

The environmental impact of traditional microfluidic devices encompasses various aspects, including their fabrication processes, materials, and waste generation. These devices are typically manufactured in cleanroom facilities, requiring controlled environments with stringent conditions. However, maintaining cleanrooms consumes significant energy and entails specialized infrastructure, contributing to environmental concerns.

Traditional microfluidic devices are commonly crafted from silicon, glass, and polymers. These materials’ extraction, processing, and manufacturing can have substantial environmental footprints. Silicon wafers, for instance, are resource-intensive to produce, and glass fabrication involves high-temperature processes.

The production of microfluidic devices often involves the use of chemicals, solvents, and photoresists. Disposing of these chemicals and the potential release into the environment pose pollution concerns. Additionally, the energy-intensive processes associated with traditional microfabrication techniques, such as photolithography and etching, contribute to the overall environmental impact.

Waste generation has a significant environmental impact, with manufacturing processes producing unused substrates, chemicals, and contaminated water. The proper disposal and treatment of these wastes are crucial to minimize their ecological impact. Some materials used in traditional microfluidic devices may have limited biodegradability, raising concerns about long-term environmental persistence. Moreover, the single-use nature of many microfluidic devices designed for research and diagnostics contributes to increased waste generation and challenges related to disposal.

### 10.2. Advantages of Biodegradable Paper Microfluidics

Biodegradable paper microfluidics offers several advantages in alignment with sustainable practices. Derived from renewable resources like wood pulp, biodegradable paper is an eco-friendly alternative to traditional microfluidic materials such as silicon or specific polymers. The production processes for biodegradable paper are generally less energy-intensive, resulting in a lower overall environmental impact throughout the material’s life cycle.

Cost-effectiveness is a notable advantage of biodegradable paper microfluidics, making them particularly suitable for applications where cost is a critical consideration. The ease of fabrication is another key feature as paper allows for straightforward manufacturing through cutting, folding, and printing techniques. This simplicity reduces the complexity and cost associated with manufacturing, enhancing accessibility for various applications.

One of the distinctive features of biodegradable paper microfluidics is their inherent biodegradability [249,250]. After disposal, these devices naturally break down over time, minimizing environmental impact and contributing to waste reduction. The customizability and functionalizability of paper microfluidics are additional strengths, allowing researchers to modify surfaces, integrate reagents, and tailor designs for specific assays or diagnostic tests.

The portability and simplicity of paper microfluidic devices make them well-suited for point-of-care applications, especially in remote or resource-limited settings. The reduced usage of chemicals in the fabrication process further adds to their appeal from an environmental standpoint. Biodegradable paper microfluidics offers a sustainable, cost-effective, and customizable solution with reduced ecological impact, promising them for various applications, including eco-friendly and practical diagnostic tools.

## 11. Challenges and Future Perspectives

### 11.1. Current Challenges in Paper Microfluidics

In its current state of development, paper microfluidics encounters challenges in creating intricate channels due to limitations in channel design and fluidic pathway complexity [251]. Achieving consistent and reproducible results is hindered by variations in paper properties like thickness and porosity, impacting diagnostic assay reliability. Sensitivity limitations persist, especially compared to advanced lab techniques, posing an ongoing challenge in detecting low analyte concentrations. The paper’s susceptibility to environmental conditions, such as humidity, affects reagent stability, raising concerns about long-term stability in resource-limited settings. Multiplexing, integrating multiple tests on a single paper device, is challenging due to the potential for cross-contamination. Achieving uniformity in fabrication processes like printing or cutting proves difficult, introducing variability that affects device performance and reliability. The finite shelf life of paper-based devices, attributed to potential paper and reagent degradation, prompts ongoing research to improve stability for extended storage. While excelling in qualitative analysis, paper devices face challenges in achieving precise quantitative measurements, impacting applications requiring accuracy.

### 11.2. Prospects and Potential Innovations

The potential innovations of paper microfluidic devices offer significant promise in various fields, particularly in low-cost diagnostics for point-of-care testing in resource-limited settings. The portability of paper devices is well-suited for on-site diagnostics, reducing reliance on centralized laboratories. Advancements in multiplexing capabilities on paper microfluidic devices can revolutionize testing methodologies, simultaneously detecting multiple analytes within a single test. This innovation can significantly impact healthcare and environmental monitoring, particularly in detecting pollutants and contaminants in air and water. Integrating paper microfluidics into wearable devices holds promise for developing flexible and wearable paper-based sensors, providing real-time insights into biomarkers or environmental factors. Advancements in fabrication techniques may enable more customizable designs of paper microfluidic devices, tailoring them to specific applications or user requirements and contributing to their versatility. Integration with smartphones for result readout and data analysis enhances the capabilities of paper microfluidic devices, facilitating remote monitoring and data sharing, aligning with the trend of leveraging smartphones for healthcare and diagnostic applications. Detection methods and sensitivity advancements can broaden paper microfluidics’ biological and chemical analysis applications, potentially revolutionizing healthcare, food safety, and environmental monitoring. Exploring hybrid systems that combine paper microfluidics with other technologies, such as electronic sensors or microcontrollers, could lead to sophisticated and versatile platforms with enhanced performance and functionalities, opening up new possibilities across various domains.

Ongoing research and development in paper microfluidics are expected to bring continuous innovations, expanding their applications and impact across diverse fields.

## 12. Conclusions

In conclusion, the manuscript highlights the significant contributions of paper microfluidics in addressing crucial challenges across various domains. Through innovative applications in healthcare, environmental monitoring, and food safety, paper-based sensing platforms offer versatile, cost-effective, and environmentally friendly solutions. This review underscores the potential of paper microfluidics to revolutionize diagnostics and monitoring, providing accessible tools for health assessments, pollution detection, and food quality assurance. As we move forward, continued research and development in this field promise to unlock further capabilities, paving the way for sustainable sensing solutions with widespread impact on human health and environmental well-being.

## Figures and Tables

**Table 1:** Summary of fabrication techniques for paper-based sensors.

Fabrication Techniques	Equipment and Materials Requirements	Advantages	Limitations	Ref.
Blade cutting/plotting	X-Y plotter, knife	Provides sharp features, no chemical required	Limited to 2D designs	[60,61]
Laser cutting	Laser cutter	Precise, customizable designs, suitable for large-scale production, high resolution (∼60 μm)	Requires specialized equipment and polymer films to protect the paper device from damage, may generate debris	[62,63,64,65,66]
Photolithography	UV light, heating plate, photomask, photoresists (positive/negative), mask aligner, chemicals, oxygen plasma	High resolution (∼200 μm), well-established microfabrication technique	Equipment-intensive, may involve multiple complex steps and chances of channel contamination	[67,68,69]
3D printing	3D printer, inks	Allows for complex, customized designs	Limited resolution compared to traditional microfabrication	[70,71,72,73,74,75,76]
Screen printing	Mesh screen, hot plate, transparency film, wax	Low-cost, scalable for mass production	Resolution may vary, suitable for relatively simple designs, new screens are required for different patterns	[77,78,79,80,81,82,83]
Wax printing	Hot plate, wax printer, solid wax	Simple, rapid, cost-effective, and suitable for prototyping	Limited resolution (∼550 μm), wax spread, limited channel height control, temperature sensitivity	[84,85,86,87,88,89]
Inkjet printing	Customized inkjet printer, hydrophobic ink, hot plate, and chemicals	Noncontact, suitable for rapid prototyping	Resolution may be lower than other techniques, requires multiple steps, and post-printing heating is required for some inks	[90,91,92,93,94,95,96]
Embossing	Embossing tools, adhesives, silane	Simple, flexible, suitable for rapid prototyping	Limited resolution, may affect paper integrity, susceptible to contamination	[97,98,99,100]
Origami and kirigami	Paper cutting and folding tools, adhesives	Foldable structures, flexible design, enhanced functionality, scalability	Precision challenges, design and assembly complexity, limited material compatibility	[101,102,103,104,105,106,107,108]

## Data Availability

Not applicable.
